# A new Prenylated Flavonoid **i**nduces G0/G1 arrest and apoptosis through p38/JNK MAPK pathways in Human Hepatocellular Carcinoma cells

**DOI:** 10.1038/s41598-017-05955-0

**Published:** 2017-07-18

**Authors:** Di Wang, Qian Sun, Jie Wu, Wei Wang, Guodong Yao, Tianyang Li, Xue Li, Lingzhi Li, Yan Zhang, Wei Cui, Shaojiang Song

**Affiliations:** 1 School of Traditional Chinese Materia Medica, Shenyang Pharmaceutical University, Shenyang, 110016 People’s Republic of China; 2 Key Laboratory of Structure-Based Drug Design and Discovery, Ministry of Education, Shenyang Pharmaceutical University, Shenyang, 110016 People’s Republic of China; 3 School of Life Science and Biopharmaceutics, Shenyang Pharmaceutical University, Shenyang, 110016 People’s Republic of China; 4 School of Pharmaceutical Engineering, Shenyang Pharmaceutical University, Shenyang, 110016 People’s Republic of China

## Abstract

Prenylated flavonoids have been demonstrated to possess diverse bioactivities including antitumor effects. One new, daphnegiravone D (**1**), and four known (**2**–**5**) prenylated flavonoids were isolated from *Daphne giraldii*. Their cytotoxic activities revealed that daphnegiravone D markedly inhibited the proliferation of cancer cells, but had no apparent cytotoxicity on human normal cells. Mechanistically, daphnegiravone D induced G0/G1 arrest and apoptosis, reduced the expression of cyclin E1, CDK2 and CDK4, and promoted the cleavage of caspase 3 and PARP in Hep3B and HepG2 cells. Meanwhile, daphnegiravone D increased the level of phosphorylated p38 and attenuated phosphorylated JNK. Further studies indicated that SB203580 partially reversed daphnegiravone D-induced G0/G1 arrest and apoptosis. The addition of SP600125 to both cell lines increased the cleavage of caspase 3 and PARP, but did not affect the G0/G1 arrest. Besides, *in vivo* studies demonstrated that daphnegiravone D obviously inhibited tumor growth in a nude mouse xenograft model through suppressing the proliferation of tumor cells, without significant effect on body weight or pathology characteristics. Taken together, the new compound selectively inhibited the proliferation of hepatoma cells via p38 and JNK MAPK pathways, suggesting its potential as a novel natural anti-hepatocellular carcinoma agent.

## Introduction

Hepatocellular carcinoma (HCC), one of the most common malignant tumors characterized by its high incidence and mortality rates, has increased rapidly worldwide during the past decade^1–4^. At present, there are a number of traditional and new therapeutic options for the treatment of HCC, such as liver resection, percutaneous ablation, palliative intra-arterial therapies, transplantation, and immunotherapy strategies^5, 6^. However, most patients do not have an optimal resection or a good prognosis during the healing process^6, 7^. Simultaneously, oxaliplatin and sorafenib, the main therapeutic drugs for HCC at present, remain unsatisfactory because of their side-effects and multidrug resistance^8–10^. Therefore, novel drugs with higher selectivity and more efficiency against HCC are the need of the hour.

Cell proliferation, growth, and survival are strictly controlled by the cell cycle regulatory mechanism and its dysregulation can lead to the occurrence and development of tumors^11, 12^. The complete cell cycle progression is closely modulated by multiple factors including cyclins, cyclin-dependent kinases (CDKs), and CDK inhibitors (CKIs)^13, 14^. Besides, cyclin-CDK complexes are the most common forms involved in the progression of the cell cycle. Currently, inhibitors targeting CDKs are used in clinical settings for multiple cancers^15–17^. For example, Palbociclib, a small-molecule inhibitor of CDK4 and CDK6 developed by Pfizer, is used for the treatment of breast cancer^18^. Regretfully, drugs that target cyclins or CDKs for the treatment of HCC have not been developed. Therefore, discovering novel inhibitors targeting at cell cycle-related proteins will be an important strategy to treat HCC.

Many new natural products have been discovered to fight against tumors according to recent researches^19^. *Daphne giraldii* Nitsche, used clinically for the treatment of ache, rheumatism and quadriplegia in China for thousands of years, has a number of secondary metabolites including flavonoids which exhibit significant anti-tumor activities^20–23^. In the present study, a new prenylated flavonoid was isolated from *Daphne giraldii* Nitsche and further research showed that the new compound selectively inhibited the growth of hepatoma cells *in vitro* and *in vivo* without cytotoxic effect on normal hepatic cells and had no significant effect on body weight and organ function of mice. Additionally, we found that this compound could arrest cell cycle at G0/G1 phase and induce apoptosis by regulating p38 and JNK/MAPK pathways in Hep3B and HepG2 cells, providing a new, safe, and effective agent for the treatment of HCC.

## Results

### Isolation and Identification

**Figure 1:**
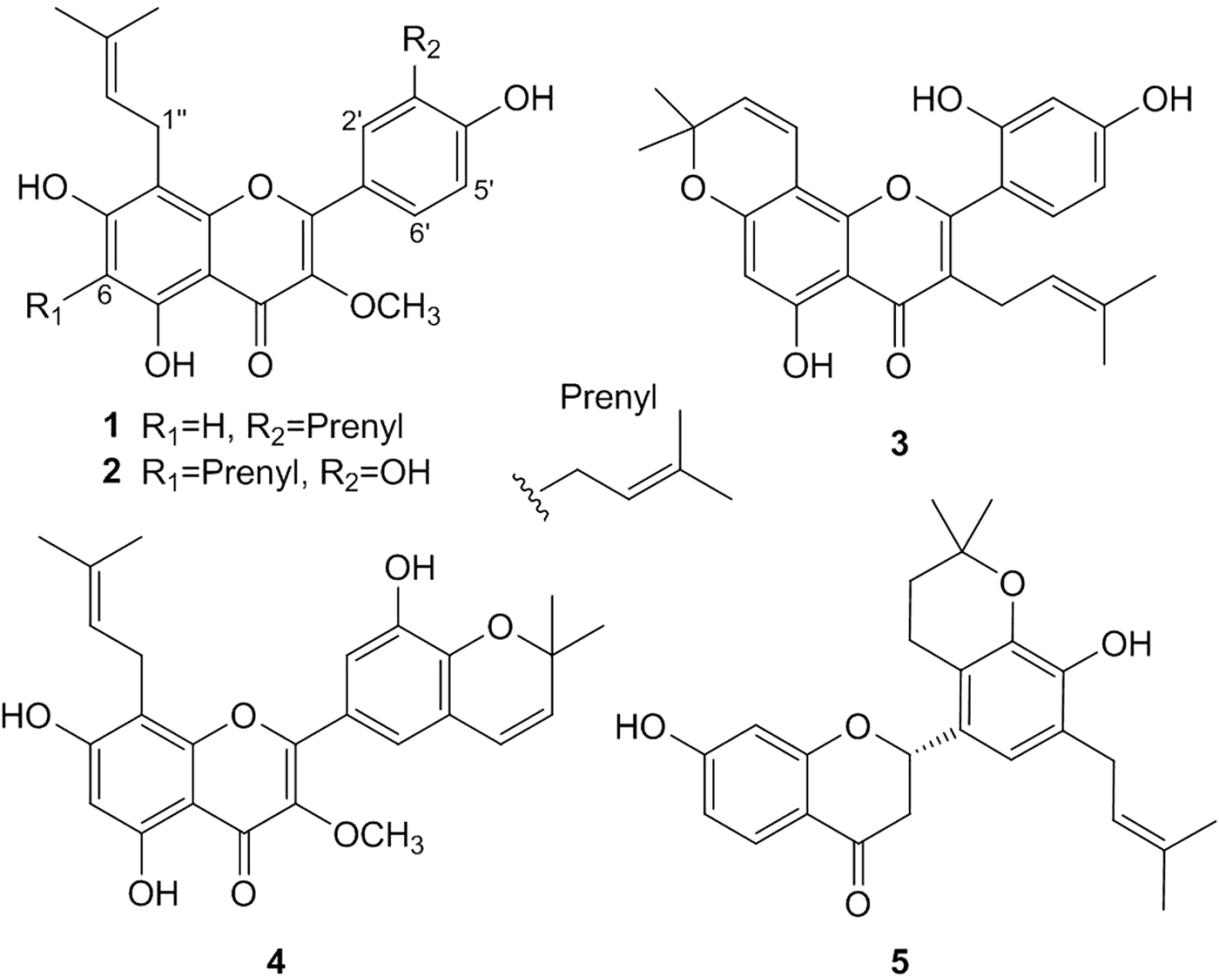
Chemical structures of compounds **1**–**5**.

Daphnegiravone D (**1**) was obtained as a yellow amorphous powder with a molecular formula of C_26_H_28_O_6_ and thirteen degrees of unsaturation as deduced from the HRESIMS ion at *m*/*z* 459.1777 [M+ Na]^+^ (calcd. 459.1778). Its UV spectrum displayed absorption bands at 272 and 357 nm. The resonances from ^1^H NMR [*δ*_H_ 12.64 (1 H, brs, OH-5)] and ^13^C NMR [*δ*_C_ 178.1 (C-4)] studies indicated that daphnegiravone D was a 5-hydroxyflavone (Table 1). Three aromatic signals at *δ*_H_ 6.28 (1 H, s), 7.80 (2 H, overlapped), 6.96 (1 H, d, *J* = 8.2 Hz) were assigned to H-6, H-2′, 6′ and H-5′, respectively, by HMBC correlations of H-6/C-5 (*δ*_C_ 158.8), C-7 (*δ*_C_ 161.5), H-2′, 6′/C-2 (*δ*_C_ 155.5) and H-5′/C-4′ (*δ*_C_ 157.9), C-6′ (*δ*_C_ 127.5) (Supplementary Figure S1.7). In addition, there were also two prenyl groups of protons at *δ*_H_ [3.37 (2 H, d, *J* = 6.6 Hz), 5.17 (1 H, t, *J* = 6.6 Hz), 1.67 (3 H, s), 1.70 (3 H, s); 3.27 (2 H, d, *J* = 7.0 Hz), 5.31 (1 H, t, *J* = 7.0 Hz), 1.60 (3 H, s), 1.67 (3 H, s)] and one methoxy signal at *δ*_H_ 3.77 (3 H, s). The positions of these substituents were confirmed by HMBC correlations. The respective cross-peaks of H-1″ (*δ*_H_ 3.37)/C-7 (*δ*_C_ 161.5), C-8 (*δ*_C_ 105.8), C-9 (*δ*_C_ 153.5) and H-1″′ (*δ*_H_ 3.27)/C-2′ (*δ*_C_ 129.2), C-3′ (*δ*_C_ 127.9), C-4′ (*δ*_C_ 157.9) suggested that two prenyl groups were linked at C-8 and C-3′. The methoxyl was situated at C-3 according to the HMBC signals of OCH_3_-3 (*δ*_H_ 3.77)/C-3 (*δ*_C_ 137.4). Moreover, the mass spectral data and downfield chemical shifts of C-7 (*δ*_C_ 161.5) and C-4′ (*δ*_C_ 157.9) supported the linkage of two additional hydroxyl groups to C-7 and C-4′, respectively. Thus, the structure of compound **1**, daphnegiravone D, was unambiguously characterized as shown (Fig. 1).

**Table 1:** ^1^H (400 MHz) and ^13^C (100 MHz) NMR data for daphnegiravone D in DMSO-*d*_6_.

Position No.	1		Position No.	1	
	*δ*_H_	*δ*_C_		*δ*_H_	*δ*_C_
2		155.5	6′	7.80, overlapped	127.5
3		137.4	1″	3.37, d, (6.6)	21.2
4		178.1	2″	5.17, t, (6.6)	122.4
5		158.8	3″		130.9
6	6.28, s	98.1	4″	1.67, s	25.4
7		161.5	5″	1.70, s	17.7
8		105.8	1″′	3.27, d, (7.0)	27.8
9		153.5	2″′	5.31, t, (7.0)	121.9
10		104.1	3″′		132.3
1′		120.8	4″′	1.60, s	25.4
2′	7.80, overlapped	129.2	5″′	1.67, s	17.5
3′		127.9	3-OCH_3_	3.76, s	59.5
4′		157.9	5-OH	12.64, brs	
5′	6.96, d, (8.2)	115.0

The five known compounds, broussoflavonol B (**2**)^24^, morusin (**3**)^25^, daphnegiravone A (**4**)^26^ and daphnegiravone C (**5**)^26^, were identified by comparison of their spectroscopic data with reported values.

### Daphnegiravone D selectively inhibits hepatocellular carcinoma cells

**Figure 2:**
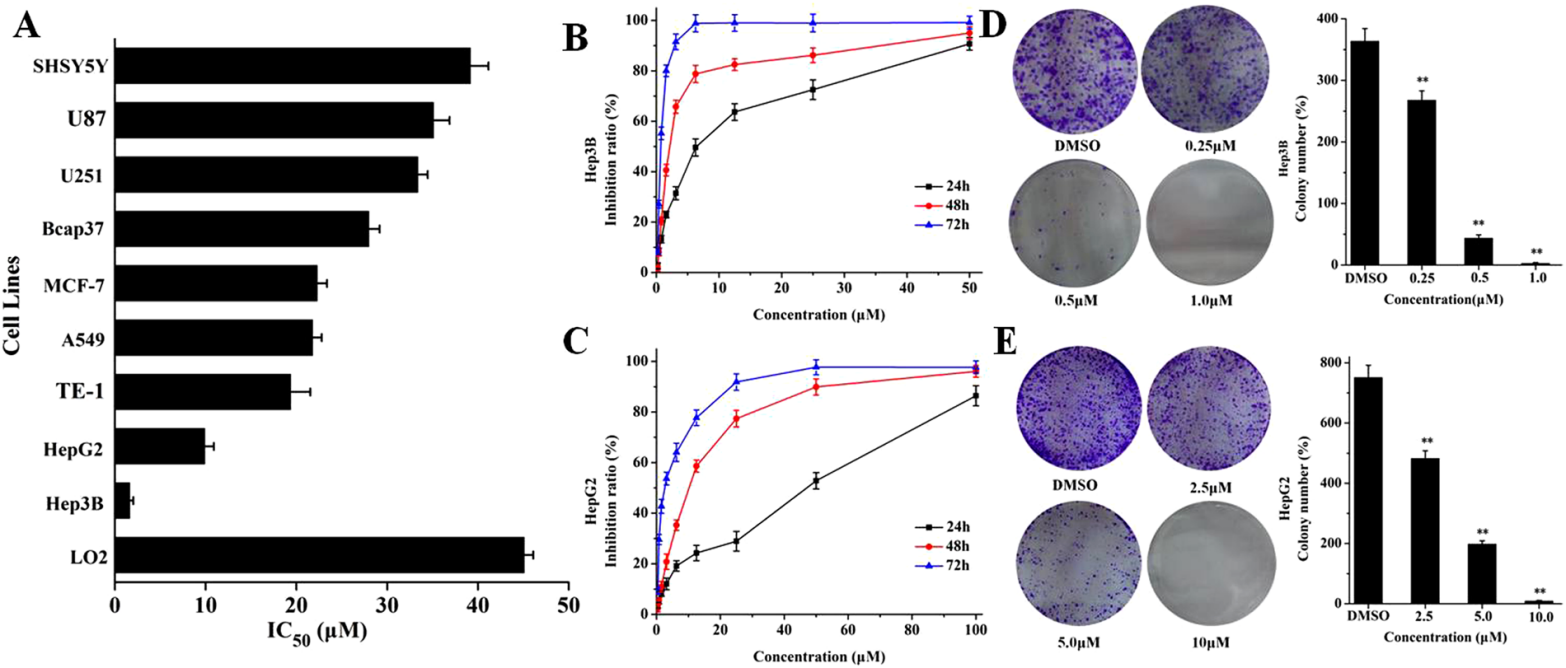
The effects of daphnegiravone D on cancer cells viability. (**A**) Selective inhibition of daphnegiravone D on nine kinds of cancer cell lines and one human normal hapatic cells. The inhibitory intensities were expressed as IC_50_. (**B**,**C**) Inhibition ratio of Hep3B and HepG2 cells treated with various concentrations of daphnegiravone D (0.2–50.0 *μ*M for Hep3B and 0.4–100.0 *μ*M for HepG2 cells) for 24, 48 and 72 h, respectively. The inhibition ratio was detected by MTT assay. (**D**,**E**) Colony formation assay demonstrated that daphnegiravone D decreased the colony formation ability in Hep3B and HepG2 cells. The number of colonies in each of the treatment groups were counted under the microscope. The statistic results are expressed as mean ± SD, n = 3, **p < 0.01 compared with control group.

Our previous studies showed that prenylated flavonoids had significant cytotoxic activities^26^, so we evaluated the *in vitro* cytotoxic potency of compounds **1**–**5** against nine human cancer cell lines and one normal hepatic cell line using MTT assay^27^ with 5-Fluorouracil (5-FU) as positive control (Table 2). The results showed that broussoflavonol B (**2**) and morusin (**3**) displayed extensive and moderate cytotoxic activities against most cancer cell lines, with IC_50_ values of about 20 *μ*M, and daphnegiravone A (**4**) and daphnegiravone C (**5**) exhibited almost no cytotoxicity for their IC_50_ values were greater than 50 *μ*M against most cell lines. Additionally, data also indicated that daphnegiravone D (**1**) exhibited potent cytotoxicity in a concentration and time-dependent manner against human hepatoma Hep3B and HepG2 cells (Fig. 2B and C) with IC_50_ values of 1.63 and 9.89 *μ*M, respectively. Also, its relatively weak cytotoxicity on other cancer cell lines and no obvious effect on normal human hepatic cells (LO2 cells with an IC_50_ value of 45.08 *μ*M) suggested that daphnegiravone D selectively inhibited hepatoma cells (Fig. 2A).

**Table 2:** Cytotoxic activities of compounds **1**–**5**.

Compounds	IC_50_ (*μ*M)									
	HepG2	Hep3B	TE-1	Bcap37	MCF-7	A549	U251	SH-SY5Y	U87	LO2
**1**	9.89 ± 1.02	1.63 ± 0.56	19.34 ± 2.30	27.97 ± 1.94	22.28 ± 2.18	21.77 ± 1.34	33.36 ± 3.03	39.15 ± 3.67	35.08 ± 3.97	45.08 ± 3.09
**2**	26.13 ± 1.89	14.22 ± 1.56	20.03 ± 2.30	42.43 ± 4.83	19.17 ± 1.76	14.98 ± 1.21	19.84 ± 1.53	13.53 ± 0.95	20.32 ± 2.08	22.41 ± 2.63
**3**	9.76 ± 0.94	14.2 ± 1.87	22.79 ± 1.79	30.98 ± 2.34	13.52 ± 2.51	23.88 ± 1.97	17.13 ± 2.22	45.36 ± 3.24	23.45 ± 2.79	28.25 ± 3.14
**4**	36.67 ± 2.21	>100	>100	>100	>100	66.76 ± 4.30	>100	74.37 ± 4.87	70.99 ± 4.36	>100
**5**	>100	55.38 ± 3.97	>100	>100	>100	20.01 ± 1.31	>100	54.77 ± 3.72	49.86 ± 3.87	>100
5FU^a^	47.09 ± 3.68	10.36 ± 1.44	26.73 ± 2.01	44.12 ± 3.82	42.34 ± 3.03	31.86 ± 2.74	42.60 ± 3.49	18.55 ± 1.38	20.15 ± 1.98	31.89 ± 3.78

^a^5FU was tested as positive control.

In order to confirm the effect of daphnegiravone D on hepatic cancer cells, clonogenic assay was performed. As shown in Fig. 2D and E, the colonies of Hep3B and HepG2 cells were dramatically reduced by daphnegiravone D in a dose-dependent manner. In summary, daphnegiravone D had a strong anti-proliferative effect on hepatoma cells.

### Daphnegiravone D results in G0/G1 arrest through downregulating cyclin E1, CDK2 and CDK4 in Hep3B and HepG2 cells

**Figure 3:**
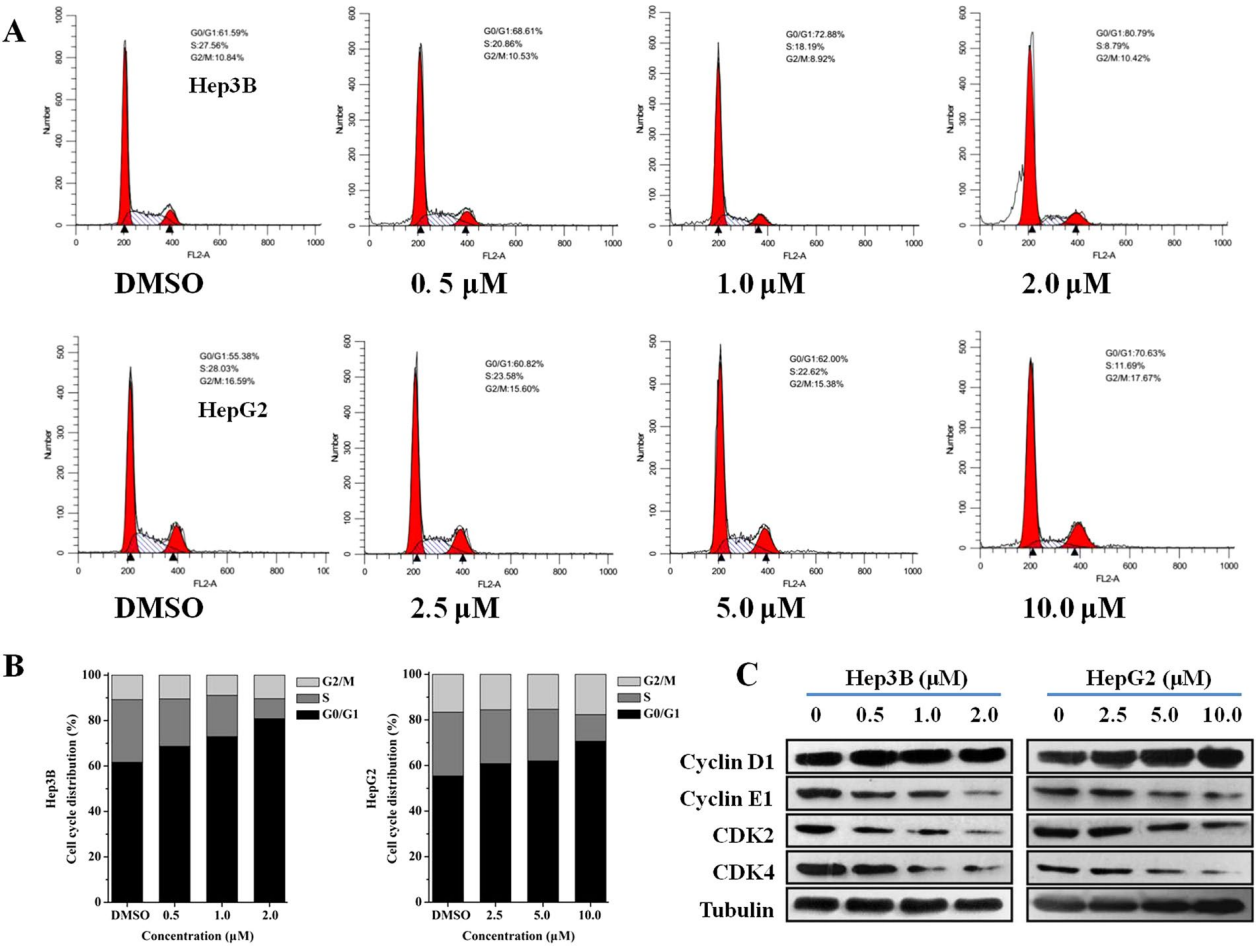
Cell cycle effects of daphnegiravone D on Hep3B and HepG2 cells. (**A**) Cells were induced with a variety of concentration (0.5–2.0 *μ*M for Hep3B and 2.5–10.0 *μ*M for HepG2 cells) respectively for 48 h, and cell cycle was analyzed by flow cytometry. (**B**) The histograms were presented to describe the effect of cell cycle. (**C**) Effects of daphnegiravone D on the expression of G0/G1-related proteins including cycline D1, cycline E1, CDK2, CDK4 were detected in both hepatoma cells. Tubulin was used as a loading control.

The cell cycle is a successive, tight and integrated physiological process that administrates proliferation, growth, and survival of cells. Abnormal cell cycle distribution can lead to decrease in proliferation, slower growth and lower survival in cancer cells^28^. To further clarify the mechanism of daphnegiravone D**-**induced inhibition of proliferation in Hep3B and HepG2 cells, propidium iodide (PI) staining was performed to determinate the DNA distribution^29, 30^. It was found that Hep3B and HepG2 cells were significantly arrested at G0/G1 phase after treatment with different concentrations of daphnegiravone D for 24 and 48 h (Fig. 3A and B; Supplementary Figure S6). Therefore, our results reveal that daphnegiravone D can induce G0/G1 phase cell arrest.

Current studies have demonstrated that cyclins regulate CDKs positively and they form complexes to drive the progression of cell cycle^31^. Cyclin D isoforms integrate with CDK4 to induce cell cycle progression through the G1 phase, while cyclin E together with its catalytic partner CDK2 to regulate the G1/S phase transition and entry into the S phase. To further explore daphnegiravone D-induced G0/G1 arrest, protein expression of cyclin D1, cyclin E1, CDK2 and CDK4 were detected using the Western blot analysis. The results indicated that daphnegiravone D decreased the levels of cyclin E1, CDK2 and CDK4 in both HepG2 and Hep3B cells in a dose-dependent manner. In contrast to cyclin E1, the expression of cyclin D1 was not significantly changed in both hepatoma cell lines (Fig. 3C). Thus, our data indicated that daphnegiravone D caused G0/G1 phase cell cycle arrest through modulating cyclin E1, CDK2 and CDK4.

### Daphnegiravone D induces apoptosis in Hep3B and HepG2 cells

**Figure 4:**
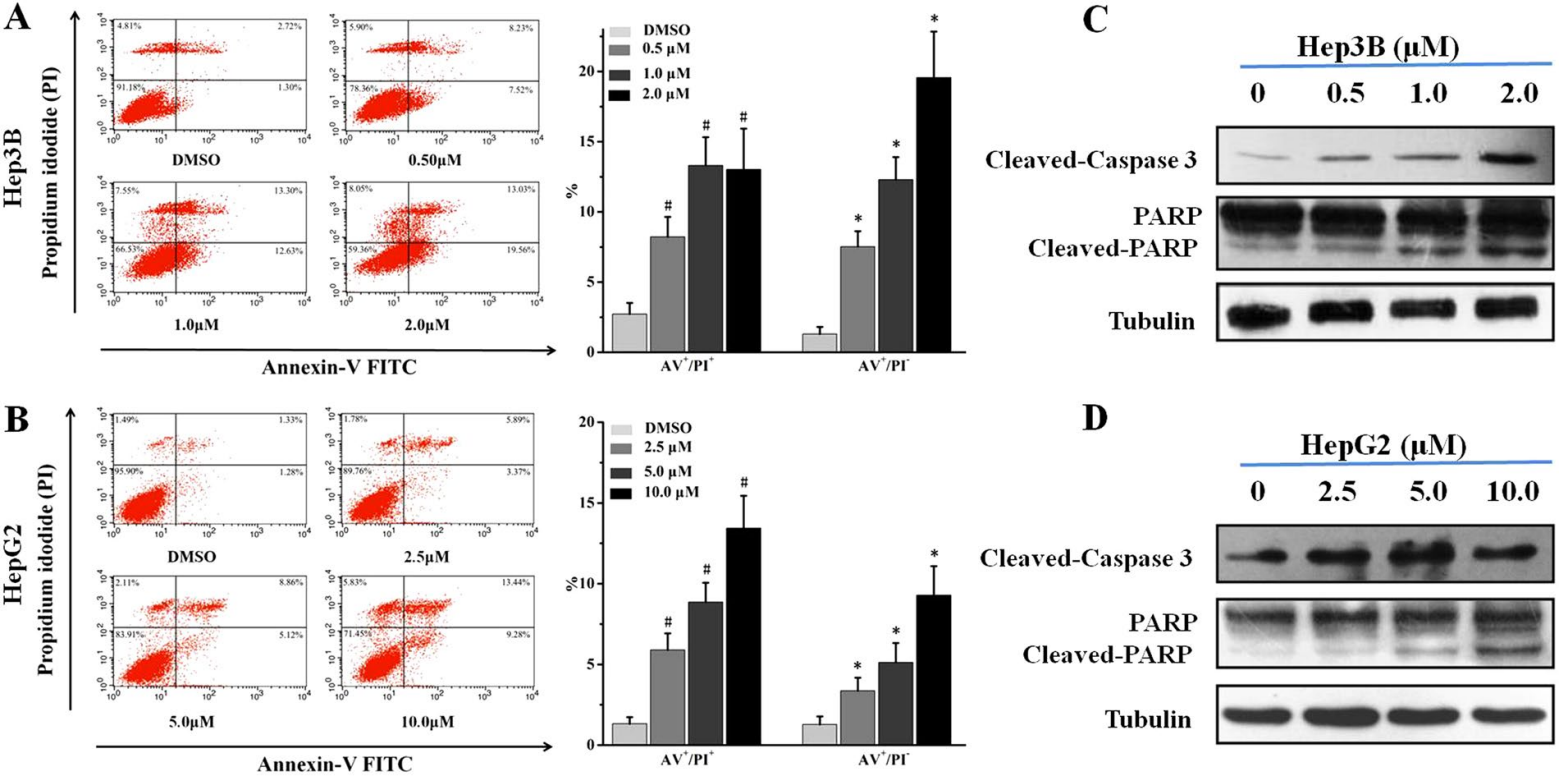
The effects of daphnegiravone D on apoptosis and the expressions of apoptosis-related proteins in Hep3B and HepG2 cells. (**A**,**B**) Cells were cultured with different concentrations of daphnegiravone D (0.5–2.0 *μ*M for Hep3B and 2.5–10.0 *μ*M for HepG2 cells) respectively for 48 h. After treatment, cells were enriched and performed with Annexin V-FITC/PI staining and analyzed by flow cytometry. (**C**,**D**) The expressions of cleaved-caspase 3 and -PARP in both hepatoma cells were assessed by Western blot after daphnegiravone D treated 48 h. Tubulin was used as a loading control. The statistical data were expressed as mean ± SD for three independent experiments. ^#^ *p* < 0.05, *p < 0.05 compared with control group.

Cell cycle arrest as a survival mechanism gives cancer cells additional time to repair their damaged DNA. If the DNA repair is delayed when the cell cycle checkpoints are abolished, the cells will undergo an apoptotic cascade^31^. Poly (ADP-ribose) polymerase (PARP), a nuclear enzyme of DNA repair that responds to DNA damage can be cleaved by  one apoptotic effector caspase 3. The cleavage of PARP means the dysfunction of the enzymatic DNA repair function, which has been researched in detail as a apoptosis marker^32^. Cell cycle analysis indicated that daphnegiravone D could induce the increase of sub-G1 peak, a characteristic of apoptosis, in Hep3B cells after treatment for 48 h (Fig. 3). Thus, we hypothesized that daphnegiravone D might induce apoptosis. Subsequently, Annexin V–FITC/PI double-staining was performed using flow cytometry to evaluate the effect of the apoptosis induced by daphnegiravone D. The results showed that AV^+^PI^−^ and AV^+^PI^+^ cells increased dramatically in a dose-dependent manner in both hepatoma cell lines after treatment with daphnegiravone D for 48 h (Fig. 4A and B), demonstrating that apoptosis was induced.

In order to further clarify the molecular mechanism of apoptosis induced by daphnegiravone D, apoptotic proteins were detected using Western blotting. The results demonstrated that daphnegiravone D increased the levels of cleaved-caspase 3 and -PARP in a dose-dependent manner (Fig. 4C and D), indicating that daphnegiravone D could induce apoptosis in Hep3B and HepG2 cells through promoting the shearing of caspase 3 and PARP.

### Daphnegiravone D inhibits cell proliferation through the p38/JNK MAPK pathways in Hep3B and HepG2 cells

MAPKs, serine/threonine kinases, can respond to extracellular stimuli and participate in intracellular signal transduction^33–35^. Considerable researches have indicated that ERK/MAPK, as a survival signal, can promote cell proliferation, differentiation and growth^36, 37^. P38/MAPK has been revealed to play a key role in DNA-damage responses, once activated, may produce cell growth inhibition and apoptosis^38^. However, cumulative reports have also shown that the activation of p38/MAPK is an essential factor for maintaining cell homeostasis^39^. Besides, highly controversially, the function of JNK/MAPK in apoptosis has been proposed. Initial biochemical research have shown that JNK/MAPK plays a pro-apoptotic role when sustained activation or dominant negative by stimulant^40^. Nevertheless, subsequent reports have indicated that JNK/MAPK also functions as anti-apoptotic to support cell survival and growth^41, 42^. These intricate functions regulated by MAPKs probably depend on the stimulus involved, the cell types and the duration of activation^39, 43^.

**Figure 5:**
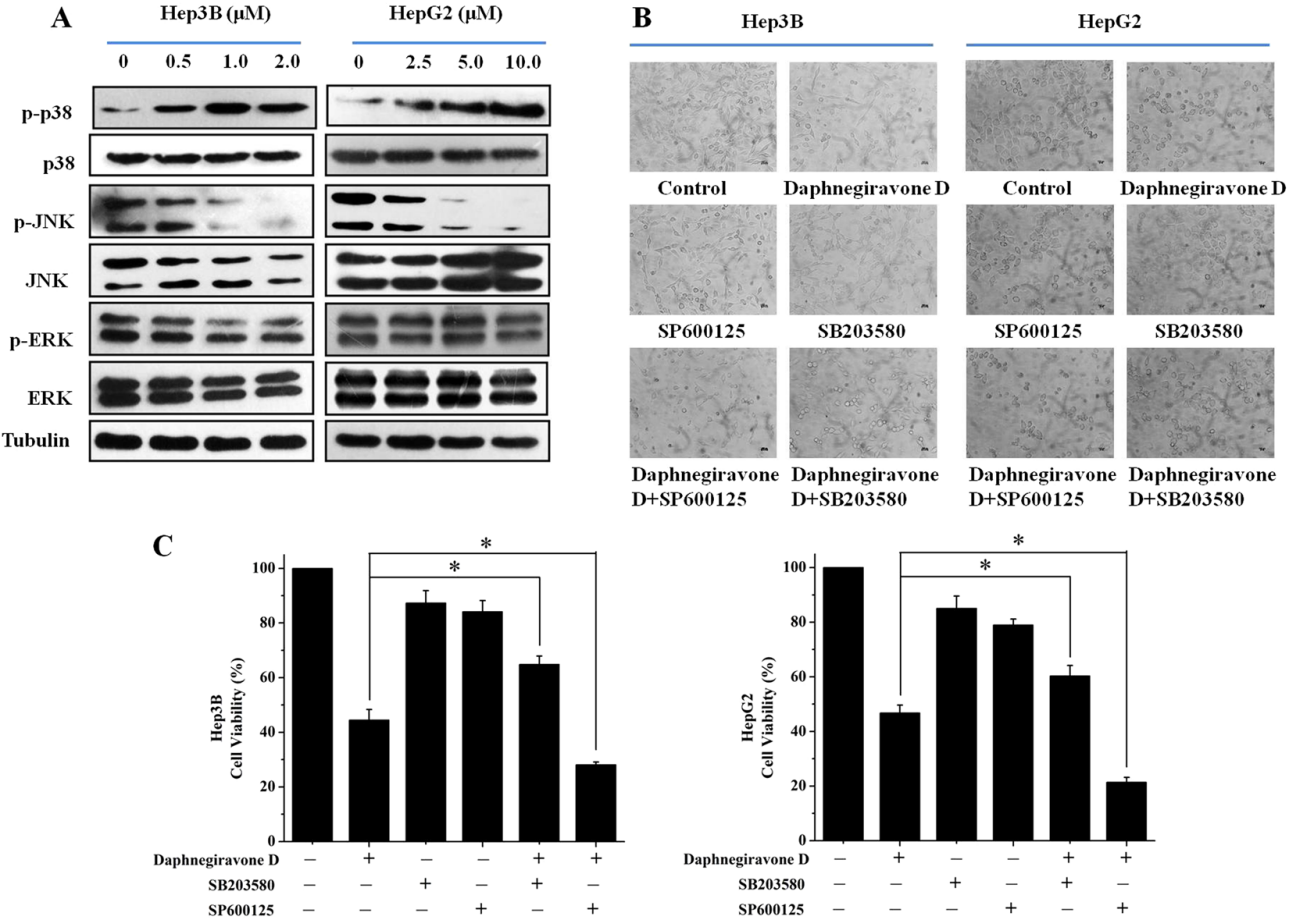
The effects of daphnegiravone D on MAPK pathway. (**A**) The effects of daphnegiravone D on the expressions of MAPK family proteins were detected using Western blot in Hep3B and HepG2 cells. Tubulin was used as a loading control. (**B**) Morphological changes were observed after Hep3B and HepG2 cells were treated with SB203580 (10 *μ*M), SP600125 (10 *μ*M) or daphnegiravone D (2 *μ*M for Hep3B cells and 10 *μ*M for HepG2 cells) for 48 h (200×). (**C**) Cell viability was detected by MTT method after treatment. The statistical data were expressed as mean ± SD for three independent experiments. *p < 0.05 compared with control group.

Since daphnegiravone D can induce G0/G1 phase arrest and apoptosis in Hep3B and HepG2 cells, we speculated that MAPK family proteins might play important roles in the inhibitory effect caused by this agent. Western blot results showed that the phosphorylation level of p38 was markedly increased following the incubation of both hepatoma cell lines with daphnegiravone D for 48 h (Fig. 5A). In contrast to the increase of phosphorylated p38/MAPK, the level of phosphorylated JNK was dramatically reduced in a dose-dependent manner after treatment with daphnegiravone D. However, there was no distinct change in the phosphorylated status of ERK. To confirm the role of p38/MAPK and JNK/MAPK pathways in the daphnegiravone D caused inhibitory effect on hepatoma cell, SB203580 (a p38/MAPK specific inhibitor) and SP600125 (a JNK/MAPK specific inhibitor) were applied. HepG2 and Hep3B cells were pretreated with 10 *μ*M SB203580 or SP600125 for 1 h followed by treatment with 10 *μ*M and 2 *μ*M daphnegiravone D for 48 h, respectively. Microscope observations showed that the morphology and number of cells were obviously affected after treatment with daphnegiravone D (Fig. 5B). At the same time, we found that SB203580 markedly antagonized the anti-proliferative effect of daphnegiravone D. Meanwhile, the anti-proliferative effect of daphnegiravone D was significantly reinforced by SP600125. MTT assay also showed that the cell viability after treatment with daphnegiravone D was clearly reduced, and this effect was abolished by SB203580 and exacerbated by SP600125, respectively (Fig. 5C). The above data suggest that p38/MAPK and JNK/MAPK pathways may be involved in the anti-proliferation effect of daphnegiravone D.

### Effect of p38/JNK MAPK inhibitors on daphnegiravone D-induced cell cycle arrest in Hep3B and HepG2 cells

**Figure 6:**
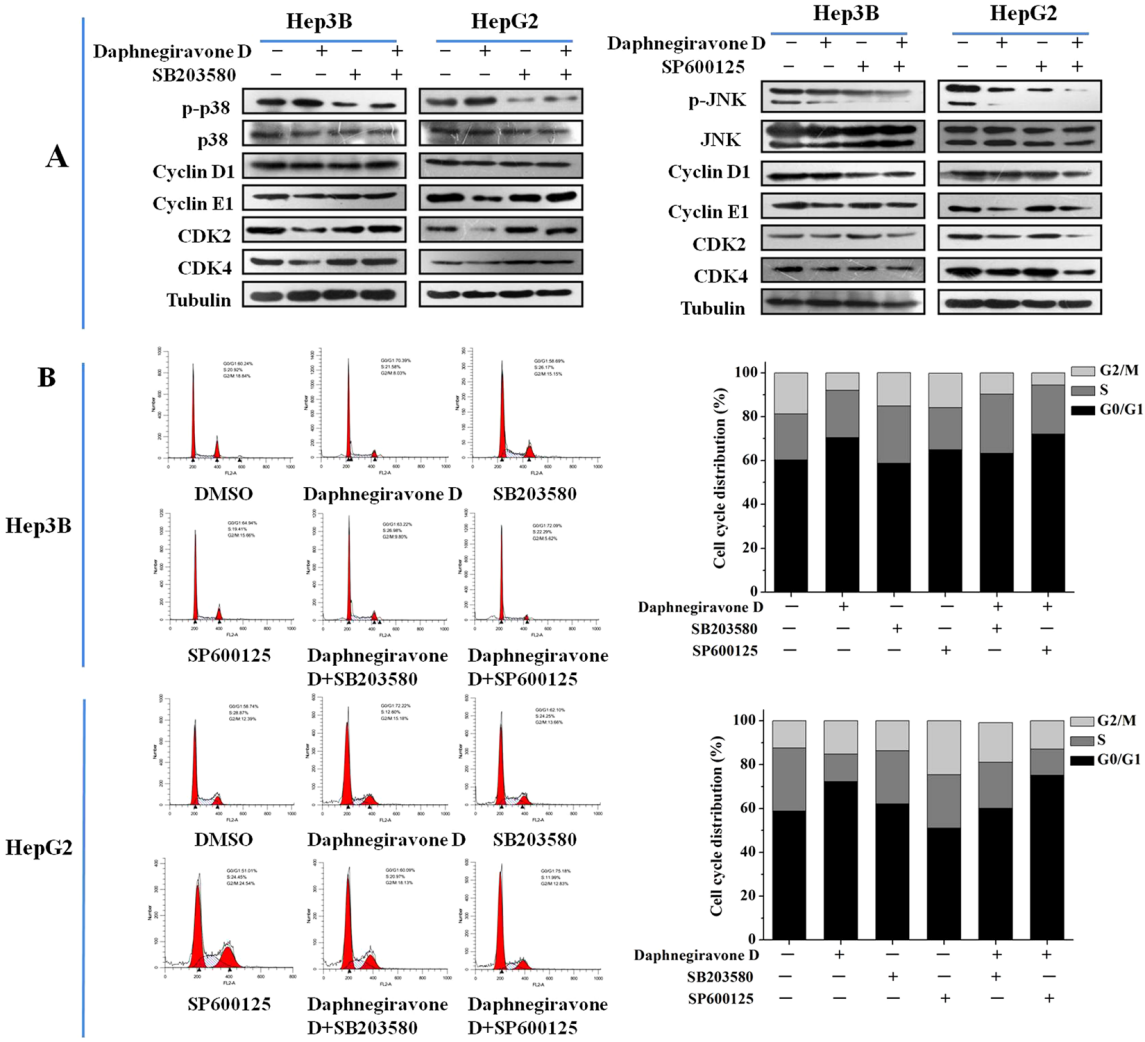
Effects of p38/JNK MAPK inhibitors on the G0/G1 phase arrest induced by daphnegiravone D in HepG2 and Hep3B cells. Cells were pretreated with SB203580 (10 *μ*M) and SP600125 (10 *μ*M) for 1 h prior to daphnegiravone D treatment (2 *μ*M for Hep3B cells and 10 *μ*M for HepG2 cells) for 48 h. (**A**) The expression levels of cycline D1, cycline E1, CDK2, CDK4 were detected by Western blot. Tubulin was used as a loading control. (**B**) Cell cycle distribution was detected by flow cytometry after treatment in both cancer cells.

Next, we further investigated the correlation between p38/JNK MAPK and the G0/G1 phase arrest induced by daphnegiravone D. Western blotting analysis showed that the inhibitory effects on cyclin E1, CDK2 and CDK4 expression in daphnegiravone D-treated cells were reversed by p38/MAPK inhibitor (Fig. 6A), and there was no obvious difference in the expression of cyclin E1, CDK2 and CDK4 between daphnegiravone D treatment alone and daphnegiravone D pretreated with SP600125 in Hep3B and HepG2 cells. These results suggest that p38/MAPK, but not JNK/MAPK, plays a crucial role in G0/G1 phase arrest induced by daphnegiravone D. To further confirm this result, flow cytometry was performed. As shown in Fig. 6B, G0/G1 arrest was roused after stimulation of HepG2 and Hep3B cells with daphnegiravone D. Simultaneously, the marked reversal of G0/G1 arrest induced by daphnegiravone D was observed following co-treatment with SB203580 and daphnegiravone D, and there was no statistical significant difference between the daphnegiravone D group and the daphnegiravone D plus SP600125 group in terms of the G0/G1 phase population (*P* > 0.05), which were consistent with the Western blotting results. Taken together, our data demonstrates that daphnegiravone D induces G0/G1 phase cell cycle arrest in a p38/MAPK but not JNK/MAPK dependent manner.

### Effect of p38/JNK MAPK inhibitors on daphnegiravone D-induced apoptosis of Hep3B and HepG2 cells

**Figure 7:**
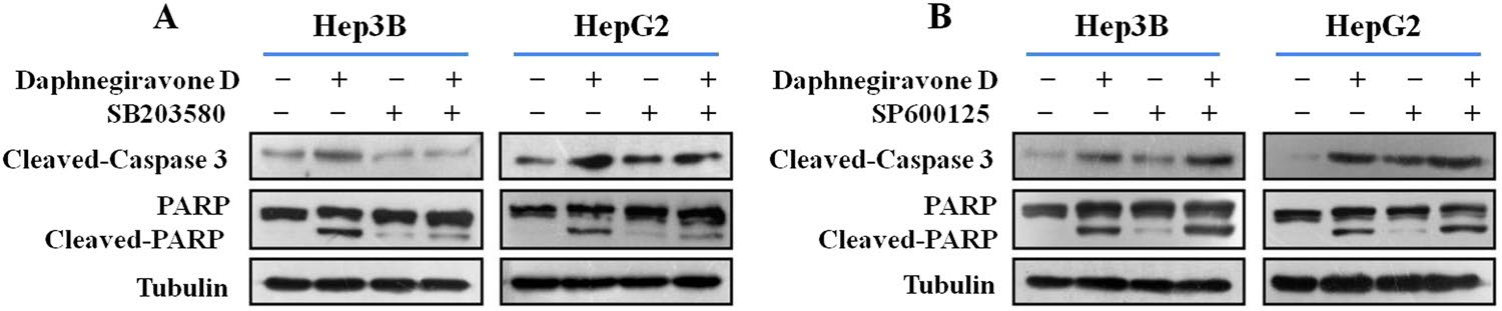
Effects of p38/JNK MAPK inhibitors on apoptosis induced by daphnegiravone D in HepG2 and Hep3B cells. Cells were pretreated with SB203580 (10 *μ*M) and SP600125 (10 *μ*M) for 1 h prior to daphnegiravone D treatment (2 *μ*M for Hep3B cells and 10 *μ*M for HepG2 cells) for 48 h. (**A**,**B**) The expression levels of cleaved-caspase 3 and -PARP were detected by Western blot in both hepatoma cells. Tubulin was used as a loading control.

In order to further confirm the roles of p38 and JNK MAPK in daphnegiravone D-induced apoptosis, cells were pretreated with p38 or JNK MAPK inhibitor. The results showed that caspase 3 was significantly activated and the cleavage of PARP was also promoted by daphnegiravone D, while these effects were clearly abolished by SB203580 in both hepatoma cell lines (Fig. 7). Meanwhile, the enhanced effects of activated caspase 3 and cleaved-PARP were observed following combined treatment with SP600125 and daphnegiravone D. These results demonstrate that daphnegiravone D can promote the activation of caspase 3 and the cleavage of PARP through accelerating the phosphorylation of p38/MAPK to trigger the signal transduction of the apoptosis cascade. However, JNK/MAPK plays a protective role to promote cellular survival and the resistance to apoptosis induced by daphnegiravone D in HepG2 and Hep3B cells.

### Daphnegiravone D inhibits tumor growth *in vivo*

**Figure 8:**
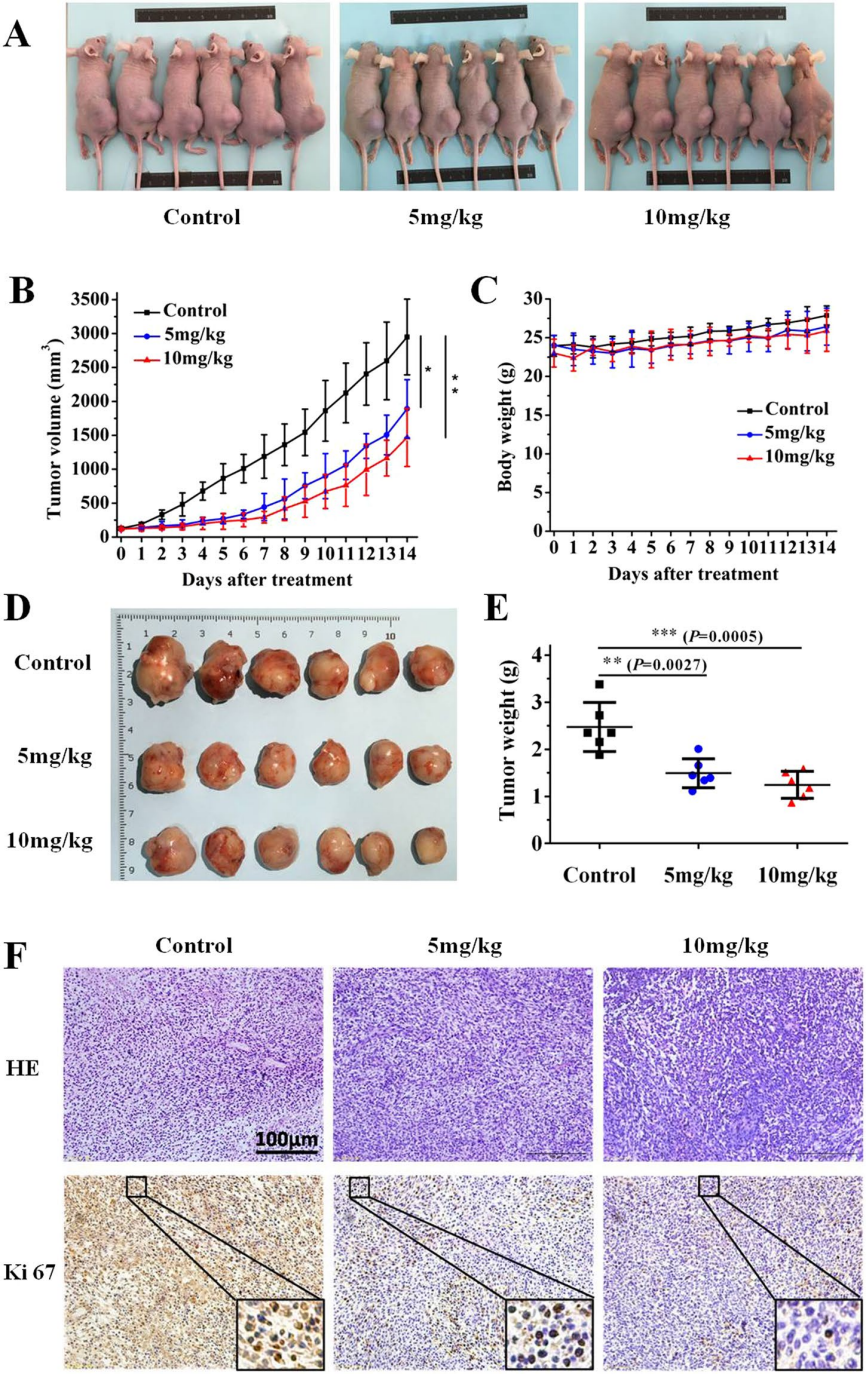
The antitumor effect of daphnegiravone D on human hepatoma Hep3B cells xenograft models. (**A**) The mice transplanted with human hepatoma Hep3B xenografts were randomly divided into three groups and given daphnegiravone D (5 mg/kg and 10 mg/kg, respectively, 1 per 2 day, i.p.) or vehicle for a period of 2 weeks. Representative photographs of the tumor xenografted nude mice. (**B**) The tumor volumes are expressed as mean ± SD (n = 6 per group). (**C**) The average body weight of each group is expressed as mean ± SD (n = 6 per group). (**D**) Representative photographs of the tumors at two weeks after daphnegiravone D treatment. (**E**) The weight of tumors harvested from mice are expressed as mean ± SD (n = 6 per group) at two weeks after daphnegiravone D treatment. (**F**) Histopathological examination of tumor tissues, as shown by H&E staining. The effects of proliferation-related biomarkers Ki-67 of Hep3B xenograft mice treated with daphnegiravone D were measured by immunohistochemistry. Scale bar, 100 *μ*m. Data are presented as means ± SD (n = 6), *P < 0.05, **P < 0.01, ***P < 0.001 versus vehicle control group.

The antitumor effect of daphnegiravone D *in vivo* was investigated in a tumor xenograft model. After tumors reached an average volume of 100 mm^3^, the nude mice bearing Hep3B xenografts were treated with daphnegiravone D every two days for two weeks. The results showed that treatment of daphnegiravone D significantly inhibited the tumor growth (Fig. 8A), the tumor volume (Fig. 8B) and tumor weight (Fig. 8D and E) were markedly decreased. The inhibition rates of the tumor volume were approximately 35.9% and 50.3% at the doses of 5 and 10 mg/kg of daphnegiravone D compared with the vehicle-administered group, respectively. Histological pathology was investigated through the H&E staining and immunohistochemical analysis of the expression of Ki-67 which is widely used as a tumor marker reflecting cell proliferation. Hep3B xenograft mice treated with daphnegiravone D showed lower proliferation capacity as indicated by Ki-67 labeling index as compared to the vehicle control (Fig. 8F).

To further test the potential side effects or toxicities of daphnegiravone D, we performed a gross evaluation and pathological study in xenograft nude mice. Daphnegiravone D-treated groups did not affect the body weight of the mice (Fig. 8C), showing that the dosage of treatment was not overtly toxic. Additionally, histopathological studies showed that there were no obvious differences in the heart, liver, spleen, lungs and kidneys among the daphnegiravone D (5 mg/kg and 10 mg/kg) treated groups and the vehicle control group, as judged by microscopic examination of tissue sections (Supplementary Figure S7), demonstrating that daphnegiravone D displayed a good safety profile. These results indicate that daphnegiravone D significantly suppress tumor growth *in vivo* without serious side effects on the mice’s growth and organ function.

## Discussion

Prenylated flavonoids, characterized by the presence of prenyl side chains on the skeleton of flavonoids, are attracting more and more attention around the world for their structural uniqueness and prominent bioactivity such as anti-tumor effect^26, 44^. However, the sources of prenylated flavonoids are very limited due to the rare occurrence in nature and the difficulty in chemical synthesis^45, 46^. *D*. *giraldii* Nitsche, a Chinese native erect shrub, belongs to genus *Daphne* (Thymelaeaceae) which has been reported to be a rich source of flavonoids compounds^23^. In the present study, five prenylated flavones including one new compound were obtained from the root bark of *D*. *giraldii* and examined for their cytotoxicity *in vitro*. The results showed that daphnegiravone D had the most potent inhibition effect on human hepatoma HepG2 and Hep3B cells with IC_50_ values of 9.89 and 1.63 *μ*M, respectively, and almost no cytotoxicity against normal human liver LO2 cells for its IC_50_ values was about 45 *μ*M, which revealed that daphnegiravone D selectively inhibits hepatocellular carcinoma cells. For prenylated flavones, the types of substituents and replacement positions may make a big difference to their antitumor potency. By comparing daphnegiravone D (**1**) and daphnegiravone A (**4**), the cyclization between the prenyl and hydroxyl moieties in ring B reduced the cytotoxicity against all tested cancer cell lines. The major structural difference between daphnegiravone D and broussoflavonol B (**2**) was the position of prenyl group and the cytotoxicity results revealed that the prenyl linked at C-3′ in ring B produced an improvement in the reduction of hepatoma cell proliferation compared with that at C-6. Combining with previous reports^47^, the similar prenylated flavone compound Kurzphenol C without a prenyl at C-3′ had weak inhibitory effect on HepG2 cells in the bioassay. Consequently, the prenyl substituent at C-3′ was speculated to be all-important to the cytotoxicity of daphnegiravone D against human hepatoma cell lines.

Previous studies revealed that the induction of cell cycle arrest and apoptosis have became the ideal strategies for the treatment of cancers^31, 48^. These processes are regulated by various intracellular cytokines, including MAPKs which play a key role in cell survival, growth, cycle, and apoptosis and have been considered as chemotherapeutic targets^44, 49, 50^. Our results showed that daphnegiravone D induce G0/G1 arrest and apoptosis of HepG2 and Hep3B cells via activating p38/MAPK and suppressing JNK/MAPK. Meanwhile, daphnegiravone D has no effect on the expression and activation of ERK/MAPK. To determine the role of p38/JNK MAPK in these physiological processes mediated by daphnegiravone D, we treated the cells with the MAPK-specific inhibitors SB203580 and SP600125. We found that daphnegiravone D-induced G0/G1 arrest was inhibited by p38/MAPK inhibitor in both hepatoma cell lines, while the JNK/MAPK inhibitors had no significant effect. Besides, apoptosis induced by daphnegiravone D was repressed by SB203580 and increased by SP600125. These results demonstrate that p38/MAPK plays an important role in daphnegiravone D-induced cell cycle arrest and apoptosis in both hepatoma cells, while JNK/MAPK plays an anti-apoptosis role in daphnegiravone D-treated cells and not associate with cell cycle arrest. In fact, several studies have reported the multi-potential regulation on signaling pathway, including MAPK pathway, of natural compound. Li *et al*.^51^ illustrated that calebin-A, a natural compound present in Curcuma longa, induced apoptosis of gastric cancer cells through increasing activity of p38 pathway and decreasing activities of JNK and ERK pathways, suggesting the different roles of MAPK pathways in apoptosis. Recently, Ke *et al*.^52^ showed that polyphylla (PP), a natural product purified from Paris polyphylla, elicited cell arrest through activating p38 pathway, simultaneously, triggered apoptosis via inhibiting ERK pathway in human tongue squamous cell carcinoma cells, indicating the multi-potential of natural compound and complexity of MAPK pathways. Therefore, it is possible that natural compound daphnegiravone D owns multiple regulation potential on MAPK pathway to execute its anti-tumor effect.

One of the advantages of natural compound for treatment with tumor is the relative low toxicity. Here, we found that daphnegiravone D showed a selective inhibition to hepatoma cells as compared with normal liver cells and other tumor cells. In consistent with the *in vitro* data, daphnegiravone D didn’t exhibit a gross toxicity to the xenograft mice models in a pharmacological dose. Furthermore, pathological detection showed that daphnegiravone D had no significant effect on histological morphology of heart, liver, spleen, lung and kidney. The above results demonstrate that daphnegiravone D displays a good toxicity profile at the pharmacological dose.

**Figure 9:**
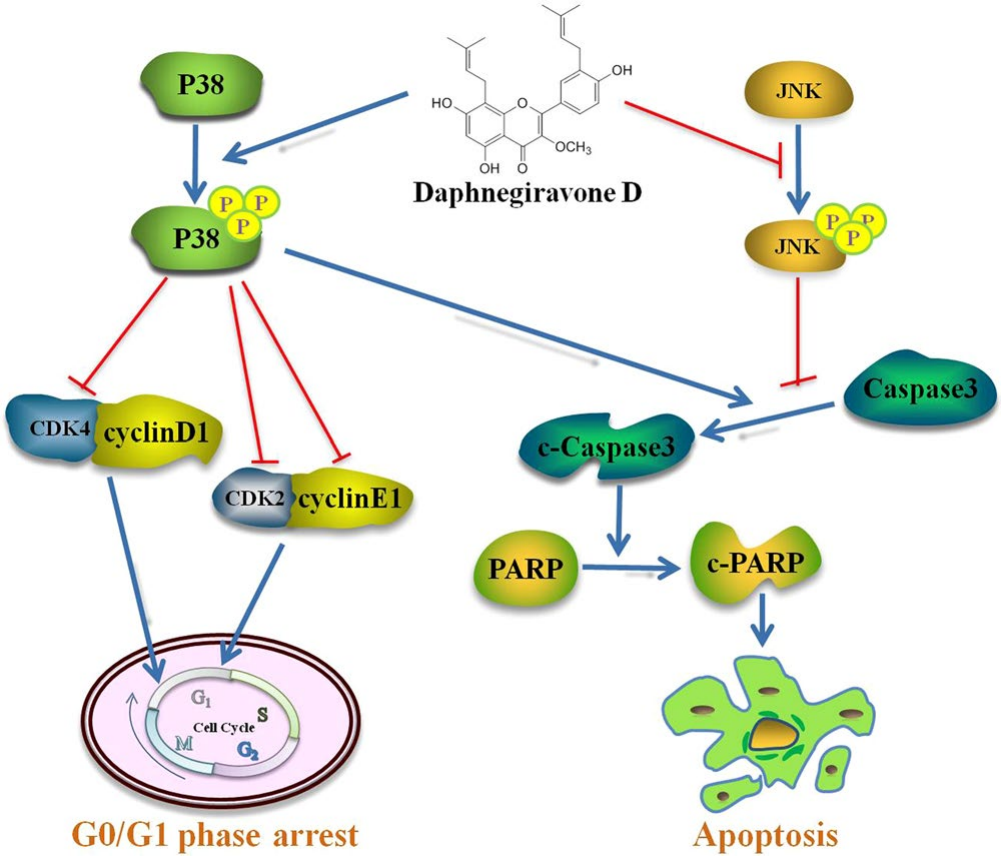
Schematic form of the proposed mechanisms of G0/G1 arrest and apoptosis induced by daphnegiravone D on hepatoma cells. Daphnegiravone D promoted the phosphorylation of p38/MAPK, down-regulation the expression of cyclin E1, CDK 2 and CDK4, induced G0/G1 arrest in Hep3B and HepG2 cells. Besides, p38/MAPK could promote apoptosis via accelerating the cleavage of caspase 3 in daphnegiravone D-treated cells. Furthermore, daphnegiravone D attenuated phosphorylated JNK, promoted the cleavage of caspase 3 and PARP, induced apoptosis in both hepatoma cells.

In summary, the present work show that daphnegiravone D markedly suppress the proliferation and survival of hepatoma cells, and induce G0/G1 arrest by reducing the expression of cyclin E1, CDK2 and CDK4 in a p38-dependent manner. Additionally, treatment with daphnegiravone D could result in apoptosis accompanied by the activation of caspase 3 and the cleavage of PARP, which is associated with the activation of p38/MAPK and the inhibition of JNK/MAPK (Fig. 9). Furthermore, daphnegiravone D exhibited significant antitumor activity *in vivo* without obvious toxicity. To the best of our knowledge, this is the first report on the *in vitro* and *in vivo* antitumor efficacy of daphnegiravone D. These investigations provide a new promising natural product as a chemotherapy agent against human hepatocarcinoma.

## Methods

### General Experimental Procedures

Ultraviolet spectra were obtained on an UV-1700 spectrophotometer (SHIMADZU, Japan). The IR spectra were measured on a Bruker IFS 55 spectrophotometer (Bruker, Germany). NMR spectra data were recorded using a Bruker ARX-400 spectrometers with standard pulse sequences operating at 400 MHz for ^1^H and 100 MHz for ^13^C, respectively, and chemical shifts were expressed with ppm (*δ*). HRESIMS was detected on a Bruker Micro Q-TOF spectrometer. Column chromatography was performed using MCI gel (CHP20P, 75–150 *μ*m; Mitsubishi Chemical Industries Ltd. Japan), C18 reversed-phase (RP) silica gel (60–80 mm; Merck, Germany), and silica gel (100–200 and 200–300 mesh; Qingdao Marine Chemical Inc. China). Precoated silica gel GF254 plates (Yantai Zifu Chemical Group Co. China) were used for TLC analysis. Semipreparative high performance liquid chromatography was carried out on a Shimadzu LC-6AD HPLC equipped with a SPD-20A UV/VIS detector using a YMC Pack ODS-A column (250 × 10 mm, 5 *μ*m).

### Plant and Reagents Material

The stem and root bark of *D*. *giraldii* were purchased from Anguo herbal medicine market (HeBei, China) in May 2012. The plant was identified by Prof. Jin-Cai Lu, (Traditional Chinese Materia Medica, Shenyang Pharmaceutical University), and a voucher specimen (No. DG-20120515) was archived in the department.

4,5-dimethylthiazol-2-yl-2,5-diphenyl tetrazolium bromide (MTT) and 5-Fluorourcil (5-FU) was purchased from Sigma-Aldrich Co., Ltd (St. Louis, USA). SB203580 (p38 inhibitor) and Sp600125 (JNK inhibitor) were provided from Selleck Chemicals (Houston, USA). RPMI 1640, Dulbecco’s modified Eagle’s medium (DMEM) and Antibiotics (100 U/mL penicillin, 100 *μ*g/mL streptomycin) were purchased from HyClone, Inc (Utah, USA). Fetal bovine serum (FBS) was purchased from Life Technologies Corporation (Gibco BRL, Grand Island, USA). Annexin-FITC Apoptosis Detection Kit was purchased from KeyGen Biotech. Co., Ltd (Nanjing, China). Propidium Iodide (PI) Cell-cycle Detection Kit was purchased from Beyotime Institute of Biotechnology (Shanghai, China). Anti-Cyclin D1, Cyclin E1, CDK2, CDK4, Cleaved-caspase 3 antibodies were acquired from abcam (Cambridgeshire, England). Anti-PARP, p38, p-p38, JNK, p-JNK, ERK, p-ERK antibodies were obtained from Cell Signaling Technology, Inc (Boston, USA). Goat anti-rabbit IgG horseradish peroxidase (HRP) antibodies were purchased from Zhongshan Goldenbridge (Beijing, China). Bicinchoninic acid (BCA) protein assay kit was purchased from Thermo Fisher Scientific Co., Ltd (Shanghai, China). Protease inhibitor cocktail was purchased from Roche (Roche, Mannheim, Germany).

### Extraction and Isolation

The dried stem and root bark of *D*. *giraldii* (18.8 kg) were extracted with 95% EtOH for a week at room temperature. The concentrated extract (986 g) was partitioned by vacuum liquid chromatography (VLC) on silica gel eluted with CH_2_Cl_2_/MeOH (100:1, 30:1, 15:1, and 0:100 v/v) to yield fractions A–D. Fraction B (280 g) was chromatographed over silica gel column with PE/EtOAc (50:1, 20:1, 10:1, and 0:100 v/v) as the eluent to give B1–B5. Subsequently, fraction B3 (65.3 g) was chosen to subject MCI CHP20P using MeOH/H_2_O (40:60, 60:40, 80:20, and 100:0 v/v), and then submitted to ODS column using MeOH/H_2_O (50:50, 60:40, 70:30, 80:20, and 100:0 v/v) to produce B3c-1–B3c-5. Subfraction B3c-3 (8.2 g) was applied to silica gel (PE/EtOAc 20:1, 10:1, 5:1, 3:1, 1:1, and 0:1 v/v) to obtain ten fractions B3c-3-1–B3c-3-10. The sixth fraction B3c-3-6 (401 mg) was further separated by ODS (70% MeOH in H_2_O) and semipreparative HPLC (45% MeCN in H_2_O, 3 mL/min) to afford **5** (3.1 mg, *t*_R_ 10.9 min), **4** (4.8 mg, *t*_R_ 13.0 min), **3** (10.0 mg, *t*_R_ 13.7 min) and **1** (85.3 mg, *t*_R_ 16.6 min). By using the similar methods of semipreparative HPLC (80% MeOH in H_2_O, 3 mL/min), fraction B3c-3-9 (288 mg) yielded compound **2** (25.0 mg, *t*_R_ 44.5 min)

*Daphnegiravone D* (***1***): yellow, amorphous powder; UV (MeOH) *λ*_max_ (log *ε*) 272 (3.84), 357 (3.69) nm; IR (KBr) *ν*_max_ 3423, 3195, 2967, 2920, 2851, 1652, 1552, 1424, 1361, 1286, 1189 cm^−1^; ^1^H and ^13^C NMR data, see Table 1; HRESIMS *m*/*z* 459.1777 [M+ Na]^+^ (calcd for C_26_H_28_O_6_Na, 459.1778).

### Cell Culture

HepG2 and Hep3B (human hepatoma cells), Bcap37 and MCF-7 (human breast cancer cells), TE-1 (human esophageal cancer cells), A549 (human lung carcinoma cells), U251 and U87 (human glioma cells), were cultured in Dulbecco’s modified Eagle’s medium (DMEM) supplemented with 10% fetal bovine serum (FBS) and 1% penicillin/streptomycin (10000 U/mL penicillin, 10000 *μ*g/ml streptomycin). SH-SY5Y (human neuroblastoma cells), LO2 (human normal liver cell) were grown in RPMI 1640 containing 10% FBS and 100 g/ml penicillin, 100 g/ml streptomycin. HepG2, Hep3B, MCF-7, TE-1, A549, U87, U251 and SHSY5Y cells were obtained from the American Type Culture Collection (Manassas, VA, USA). Bcap37 and LO2 cells were purchased from the Cell Bank of Chinese Academy of Sciences (Shanghai, China). All cells were incubated in a humidified atmosphere containing 5% CO_2_ at 37 °C.

### Cytotoxicity Assay

Cells seeded in 96-well plates with 5 × 10^3^ cells/well were adhered overnight and incubated with vehicle control (DMSO) or diverse concentrations of compounds for 48 h. After treatment, 20 *μ*L of MTT (5 mg/mL) was added to each well and incubated for another 4 h at 37 °C. Then, 150 *μ*L of DMSO/well was appended after removed the supernatant to dissolve the formed purple formazan crystals and the color absorbance was measured at 490 nm using a microplate reader (Molecular Devices, Thermo, CA, USA). The inhibition rate (%) was calculated as (A_490(control)_ − A_490(drug groups)_)/(A_490(control)_ − A_490(blank)_) × 100 and the value of IC_50_ (the concentration that caused 50% inhibition of cell viability) was determined using SPSS 20.0. This experiment was measured in triplicate, independently.

### Clonogenic Assays

The clonogenic assay was performed to appraise the effect of daphnegiravone D on cell proliferation. Hep3B (800 cells/well) or HepG2 (4,000 cells/well) cells were seeded in 6-well plates in 2 mL complete media overnight and attachment cells were incubated with various concentrations of daphnegiravone D for two weeks. Then, cells were washed twice with PBS carefully and fixed with 4.0% paraformaldehyde for 30 min, and stained with 0.5% crystal violet at room temperature for 30 min. Colonies containing exceeding 50 cells/colony were counted under microscope. All experiments were independently performed three times.

### Cell Cycle Analysis

Flow cytometry was used to determine the cell cycle effects of daphnegiravone D on Hep3B and HepG2 cells. Cells were seeded (2 × 10^5^ cells/well) into six-well plates with complete DMEM medium for 24 h and treated with various concentrations of daphnegiravone D for 24 and 48 h. Cells were harvested after treatment, washed with cold PBS, and fixed with 70% ethanol at 4 °C overnight. Then, resuspended cell pellets in staining buffer, blended in RNase A and PI staining for 30 min at 37 °C. Cell cycle phase was investigated using a FACSCalibur flow cytometer (BD Biosciences, New Jersey, USA). The results were analyzed with FlowJo software (v7.6.5, Tree Star, Inc.).

### Annexin V-FITC Apoptosis Detection

An annexin-V FITC apoptosis detection Kit was used to determine the apoptosis effect induced by daphnegiravone D according to the manufacturer’s instructions (BD Biosciences, CA, USA). Briefly, Hep3B and HepG2 cells were plated in 6-well plates (2 × 10^5^/well) and incubated overnight. Cells were harvested and washed with PBS by centrifugation after treatment with different concentrations of daphnegiravone D for 48 h. Then, cells were resuspended in 500 *μ*L binding buffer and staining with Annexin V-FITC (10 *μ*L) and propidium iodide (5 *μ*L) at room temperature in the dark for 10 min. Thereafter, cells were analyzed by flow cytometry within 1 h after staining. 10,000 cells were acquired for each sample and data were analyzed by Cell Quest Pro software (BD Biosciences, New Jersey, USA).

### Western Blotting analysis

Cells that after treatment were harvested and lysed by radio-immunoprecipitation assay (RIPA) buffer containing protease inhibitor (PMSF, 1 mM) on ice for 30 min. Cells lysates were centrifuged at 12,000 g for 15 min at 4 °C and following the protein concentration in the supernatant was determined using a BCA protein assay kit (Thermo Scientific, Waltham, USA), then cell extracts were added SDS-PAGE sample loading buffer and denatured at 100 °C for 10 min. Equal amount of protein (30 *μ*g) were separated on a 8–15% SDS-PAGE and transferred onto PVDF membranes (0.2 *μ*m, Millipore) using an electroblotting apparatus (Bio-Rad). The membranes were blocked with 5% milk in PBS-T (PBS containing 0.1% Tween 20) for 2 h at room temperature and incubated with primary antibodies at 4 °C overnight. After washed three times with PBS-T, the membranes were incubated with horseradish peroxi-dase (HRP)-conjugated secondary antibody (1:5,000) at room temperature for 2 h, then washed again, and the immunoreactive bands were detected using ECL reagents (ThermoFisher, Waltham, MA).

### *In Vivo* Antitumor Activity

All of the animal studies and procedures were conducted in accordance with the approval and guidelines of the Shenyang Pharmaceutical University Institutional Animal Care and Use Committee (permission number: SCXK (Jing) 2014-0004). Male BALB/c nude mice at the age of six weeks were obtained from Beijing HFK Bioscience Co., Ltd (Beijing, China). Hep3B cells (1 × 10^5^ cells) were implanted subcutaneously into the right backsides of each nude mouse. After 12 days, when the tumor size reached about 100 mm^3^, the mice were randomly divided into three groups (n = 6) and intraperitoneally injected with vehicle control or daphnegiravone D (5 and 10 mg/kg body weight) every two days for two weeks. The body weight and tumor sizes were measured every day, and the tumor volume was determined by caliper measurements and calculated by the formula: 0.5236 × *L* × *W* ^2^, where *L* is the long axis and *W* is the short axis of the tumor. The experiment was terminated when the tumors volume of the control group reached about 3000 mm^3^. The mice were sacrificed, and the tumors, hearts, livers, spleens, lungs and kidneys were excised immediately to evaluate the antitumor activity and toxicity of daphnegiravone D.

### H&E Staining and Immunohistochemistry

Mouse major organs (heart, liver, spleen, lung, kidney) and tumors were isolated and fixed in 10% buffered formalin for 3 days and embedded in paraffin. The tissues were sectioned at a thickness of 4 *μ*m, and the obtained organ sections were prepared orderly by dewaxing, staining in hematoxylin and eosin-phloxine solution, then dehydrated and mounted with neutral resin. Subsequently, the tumor paraffin sections were deparaffinized, rehydrated, and subjected to antigen retrieval. Immunostaining was performed by the streptavidin-peroxidase (S-P) method (Ultra-sensitive; MaiXin, Fuzhou, China). The primary antibody was mouse anti-Ki67 monoclonal antibody (Maixin Biotech, Fuzhou, China). The peroxidase reaction was developed with 3,3-diaminobenzidine (Maixin Biotech, Fuzhou, China). Finally, the sections tissue morphology were observed using an OLYMPUS microscope.

### Statistical Analysis

Every experiment was duplicated at least three times, and the results are expressed as mean ± standard deviation. Student’s *t* -test and ANOVA were used to analyze the significances of different groups’ result and *P* < 0.05 was considered as statistically significant.

## Electronic supplementary material

Supplementary information
