# Supplementary material for: A new Prenylated Flavonoid induces G0/G1 arrest and apoptosis through p38/JNK MAPK pathways in Human Hepatocellular Carcinoma cells
Source: Sci Rep. 2017 Jul 18;7:5736. doi: 10.1038/s41598-017-05955-0 (PMC5515844; doi:10.1038/s41598-017-05955-0)
Supplement: Supplementary file 1 — Supplementary information [file 41598_2017_5955_MOESM1_ESM.pdf]

A new Prenylated Flavonoid induces G0/G1 arrest and apoptosis through p38/JNK MAPK pathways in Human Hepatocellular Carcinoma cells

Di Wang[1,2+], Qian Sun[1,2+], Jie Wu[1,2], Wei Wang[1,2], Guodong Yao[1,2], Tianyang Li[3], Xue Li[3], Lingzhi Li[1,2], Yan Zhang[2,4], Wei Cui[3,*], Shaojiang Song[1,2,*]

[1]*School of Traditional Chinese Materia Medica, Shenyang Pharmaceutical University, Shenyang 110016, People's Republic of China*

[2]*Key Laboratory of Structure-Based Drug Design and Discovery, Ministry of Education, Shenyang Pharmaceutical University, Shenyang 110016, People's Republic of China*

[3]*School of Life Science and Biopharmaceutics, Shenyang Pharmaceutical University, Shenyang 110016, People's Republic of China*

[4]*School of Pharmaceutical Engineering, Shenyang Pharmaceutical University, Shenyang, 110016, People's Republic of China*

*Corresponding author. Tel: +86 24 23986510 (S. J. Song), +86 24 23986265 (W. Cui).

E-mail addresses: songsj99@163.com. (S. J. Song), cuiwei_syphu@126.com (W. Cui).

[+]these authors contributed equally to this work

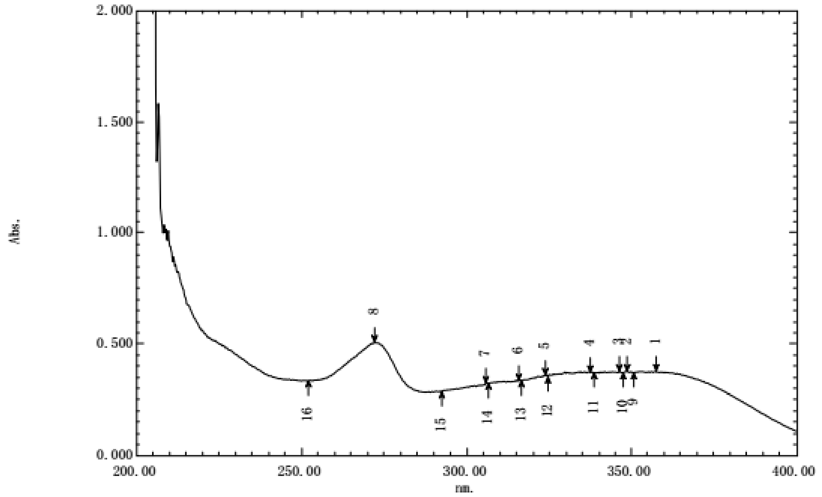

测定属性
波长范围 (nm.): 200.00到400.00
扫描速度: 高速
采样间隔: 0.2
自动采样间隔: 停用
扫描模式: 单一的

试样准备属性
重量: 0.3
体积 : 10
稀释:
光程长: 407
附加信息:

仪器属性
仪器类型: UV-1700
测定方式: 吸收值
狭缝宽: 1.0 nm
光源改变波长: 340.8 nm
S/R 转换: 标准

附件属性
附件: 无

No.	P/V	Wavelength	Abs.	描述
1	↑	357.40	.375	
2	↑	348.80	.375	
3	↑	346.40	.374	
4	↑	337.40	.373	
5	↑	323.80	.357	
6	↑	316.00	.337	
7	↑	306.00	.325	
8	↑	272.20	.506	
9	↓	350.60	.370	
10	↓	347.40	.370	
11	↓	338.80	.368	
12	↓	324.60	.355	
13	↓	316.80	.333	
14	↓	306.80	.322	
15	↓	292.40	.284	
16	↓	252.20	.332	

Supplementary Figure S1.1 UV spectrum of daphnegiravone D

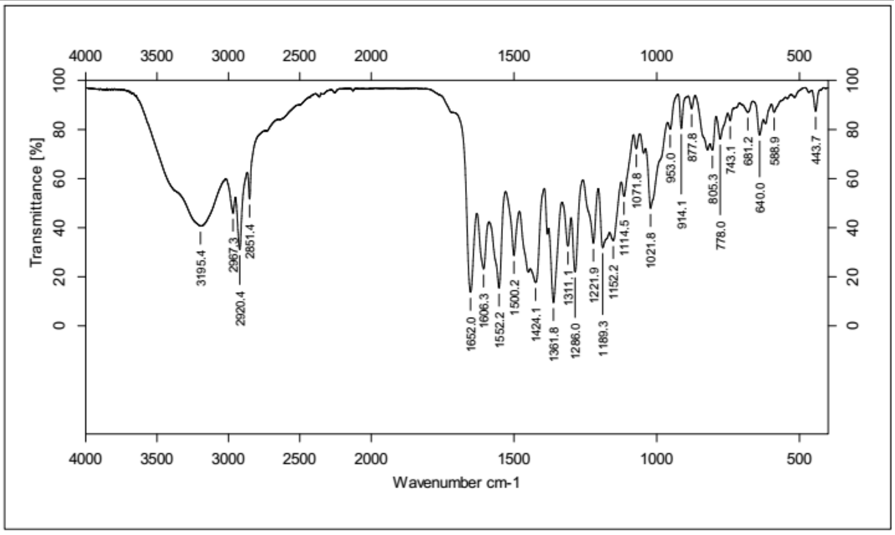

Sample : S29	Frequency Range : 3999.64 - 400.157		Measured on : 2015-12-18
Technique : Sample form	Resolution : 2	Instrument : EQUINOX55	Sample Scans : 16
Customer : Default	Zerofilling : 2	Acquisition : Double Sided,Forv	

Supplementary Figure S1.2 IR spectrum of daphnegiravone D

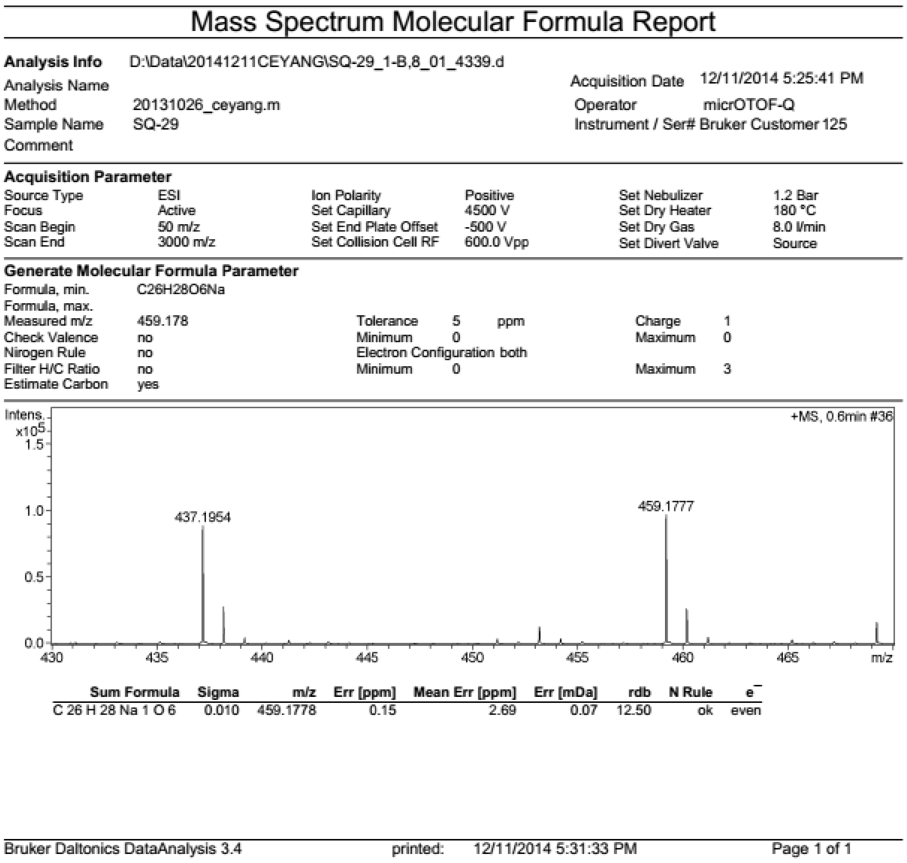

Mass Spectrum Molecular Formula Report

**Analysis Info**	D:\Data\20141211CEYANG\SQ-29_1-B,8_01_4339.d	Acquisition Date	12/11/2014 5:25:41 PM
Analysis Name			
Method	20131026_ceyang.m	Operator	micrOTOF-Q
Sample Name	SQ-29	Instrument / Ser#	Bruker Customer 125
Comment			

Acquisition Parameter

Source Type	ESI	Ion Polarity	Positive	Set Nebulizer	1.2 Bar
Focus	Active	Set Capillary	4500 V	Set Dry Heater	180 °C
Scan Begin	50 m/z	Set End Plate Offset	-500 V	Set Dry Gas	8.0 l/min
Scan End	3000 m/z	Set Collision Cell RF	600.0 Vpp	Set Divert Valve	Source

Generate Molecular Formula Parameter

Formula, min.	C26H28O6Na				
Formula, max.					
Measured m/z	459.178	Tolerance	5 ppm	Charge	1
Check Valence	no	Minimum	0	Maximum	0
Nirogen Rule	no	Electron Configuration	both		
Filter H/C Ratio	no	Minimum	0	Maximum	3
Estimate Carbon	yes				

Sum Formula	Sigma	m/z	Err [ppm]	Mean Err [ppm]	Err [mDa]	rdb	N Rule	e⁻
C 26 H 28 Na 1 O 6	0.010	459.1778	0.15	2.69	0.07	12.50	ok	even

Bruker Daltonics DataAnalysis 3.4 printed: 12/11/2014 5:31:33 PM Page 1 of 1

Supplementary Figure S1.3 HRESIMS spectrum of daphnegiravone D

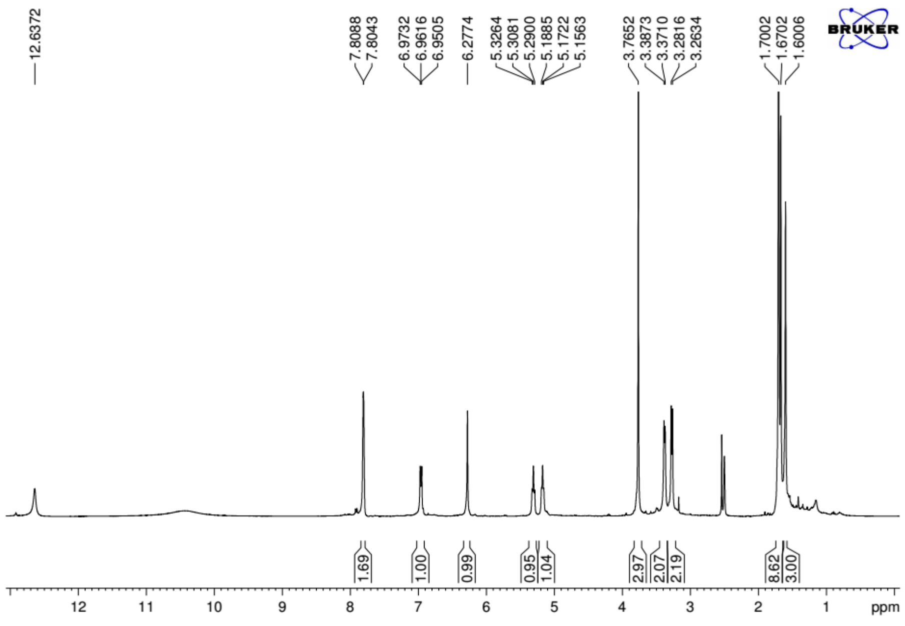

Supplementary Figure S1.4 ^{1}H NMR spectrum (400 MHz, DMSO-d_6) of daphnegiravone D

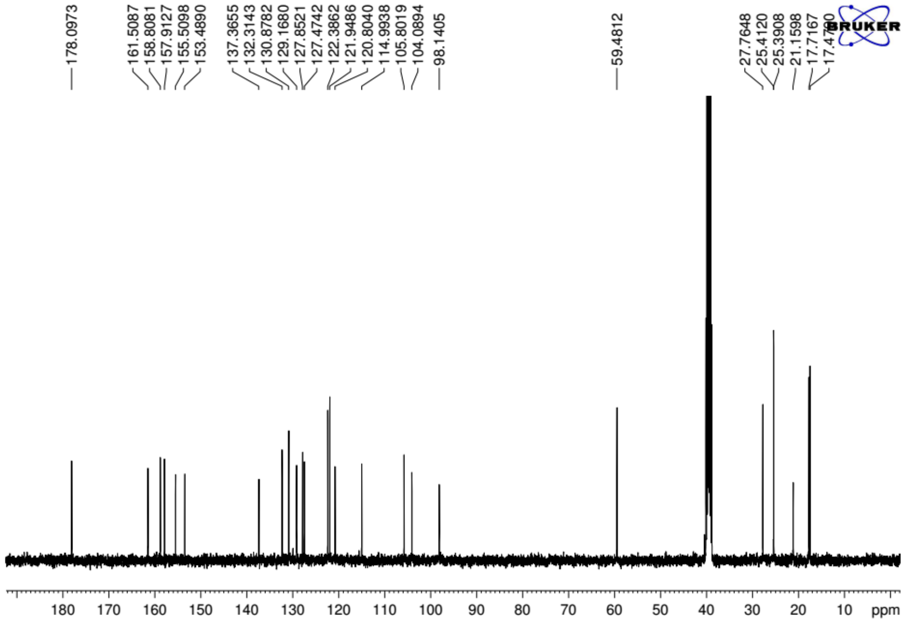

Supplementary Figure S1.5 ^{13}C NMR spectrum (100 MHz, DMSO-d_6) of daphnegiravone D

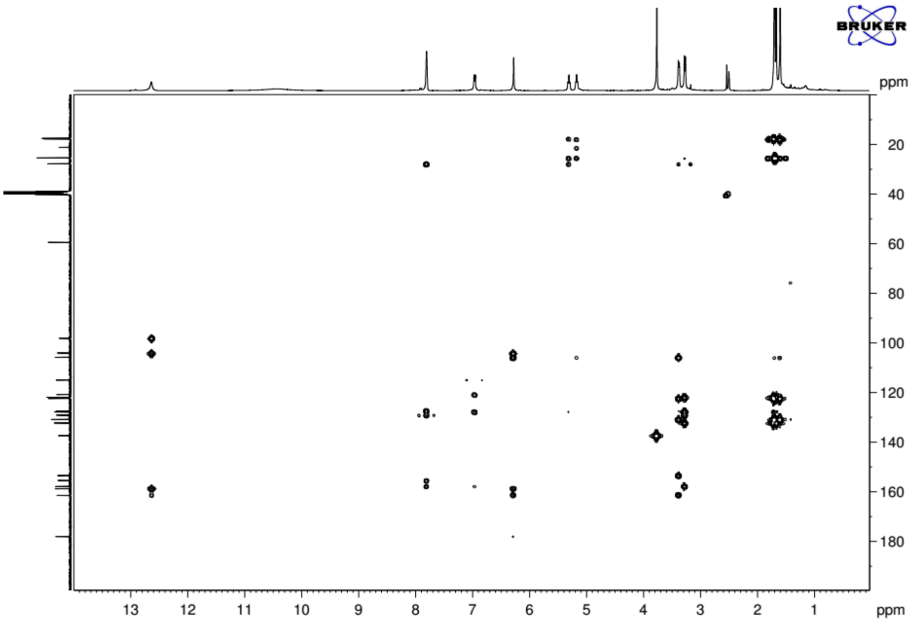

Supplementary Figure S1.6 HMBC spectrum (600 MHz, DMSO-d_6) of daphnegiravone D

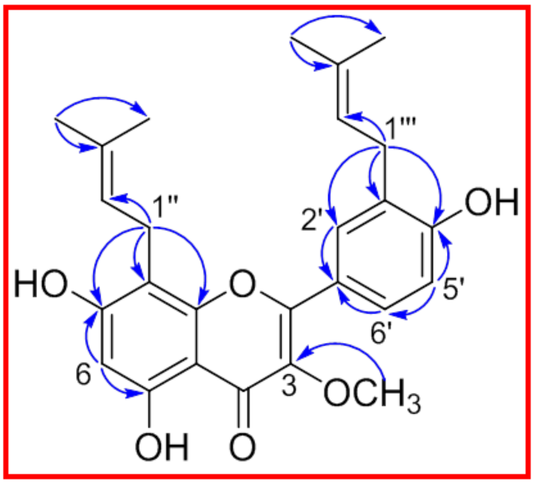

Supplementary Figure S1.7 Key HMBC correlations of daphnegiravone D.

Mass Spectrum Molecular Formula Report

Analysis Info D:\Data\20141126CEYANG\SQ-4.d

Analysis Name		Acquisition Date	11/26/2014 3:00:28 PM
Method	tune_wide_pos.m	Operator	micrOTOF-Q
Sample Name	SQ-4	Instrument / Ser#	Bruker Customer 125
Comment			

Acquisition Parameter

Source Type	ESI	Ion Polarity	Positive	Set Nebulizer	0.3 Bar
Focus	Active	Set Capillary	4500 V	Set Dry Heater	180 °C
Scan Begin	50 m/z	Set End Plate Offset	-500 V	Set Dry Gas	4.0 l/min
Scan End	3000 m/z	Set Collision Cell RF	600.0 Vpp	Set Divert Valve	Source

Generate Molecular Formula Parameter

Formula, min.	C26H28O7H				
Formula, max.					
Measured m/z	453.19	Tolerance	5 ppm	Charge	1
Check Valence	no	Minimum	0	Maximum	0
Nirogen Rule	no	Electron Configuration	both		
Filter H/C Ratio	no	Minimum	0	Maximum	3
Estimate Carbon	yes				

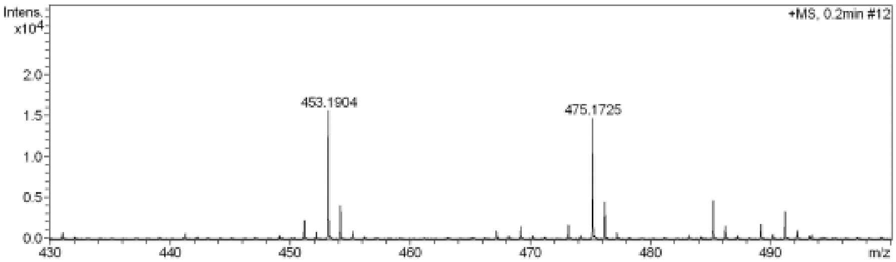

Sum Formula	Sigma	m/z	Err [ppm]	Mean Err [ppm]	Err [mDa]	rdb	N Rule	e⁻
C 26 H 29 O 7	0.016	453.1908	0.76	0.66	0.34	12.50	ok	even

Bruker Daltonics DataAnalysis 3.4 printed: 11/26/2014 3:04:03 PM Page 1 of 1

Supplementary Figure S2.1 HRESIMS spectrum of broussoflavonol B

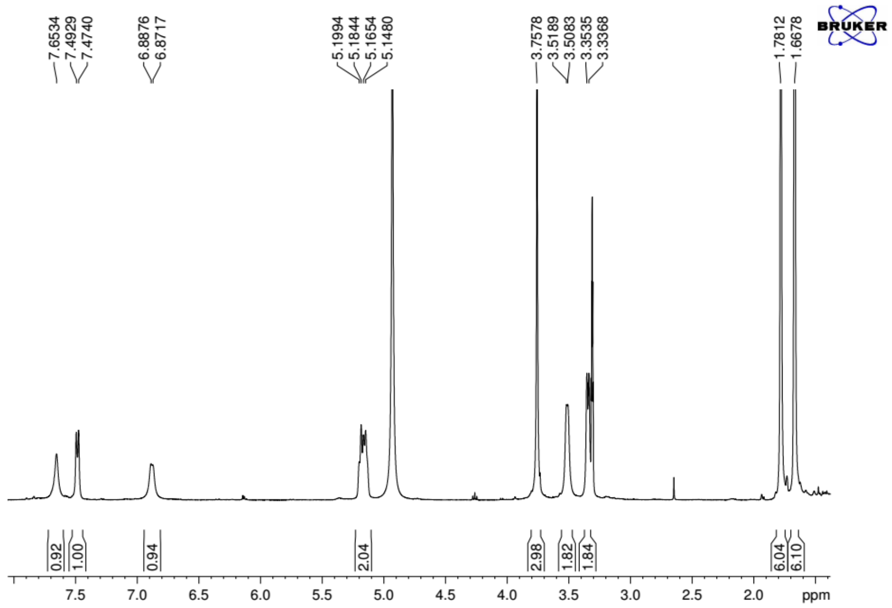

Supplementary Figure S2.2 ^{1}H NMR spectrum (400 MHz, DMSO-d_6) of broussoflavonol B

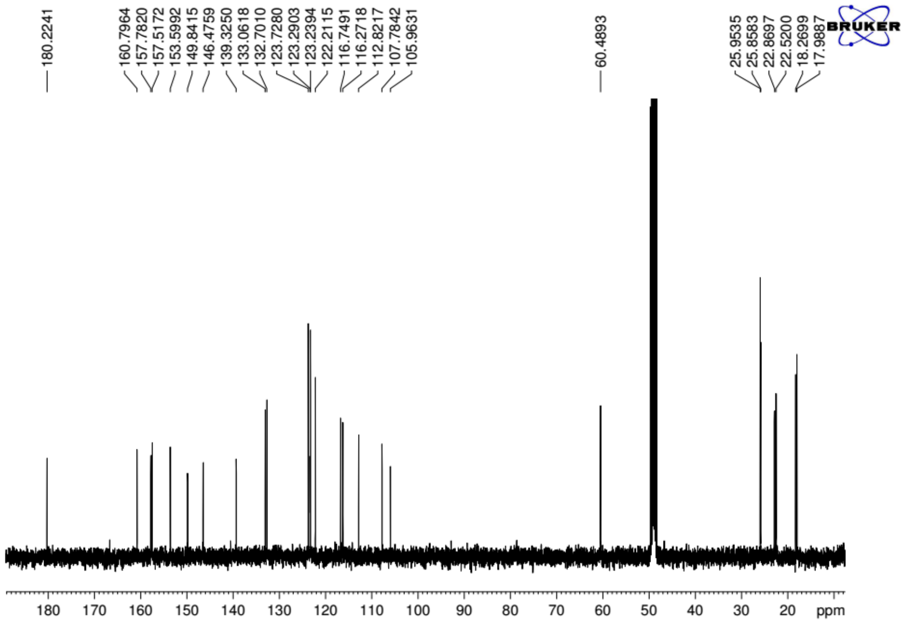

Supplementary Figure S2.3 ^{13}C NMR spectrum (100 MHz, DMSO-d_6) of broussoflavonol B

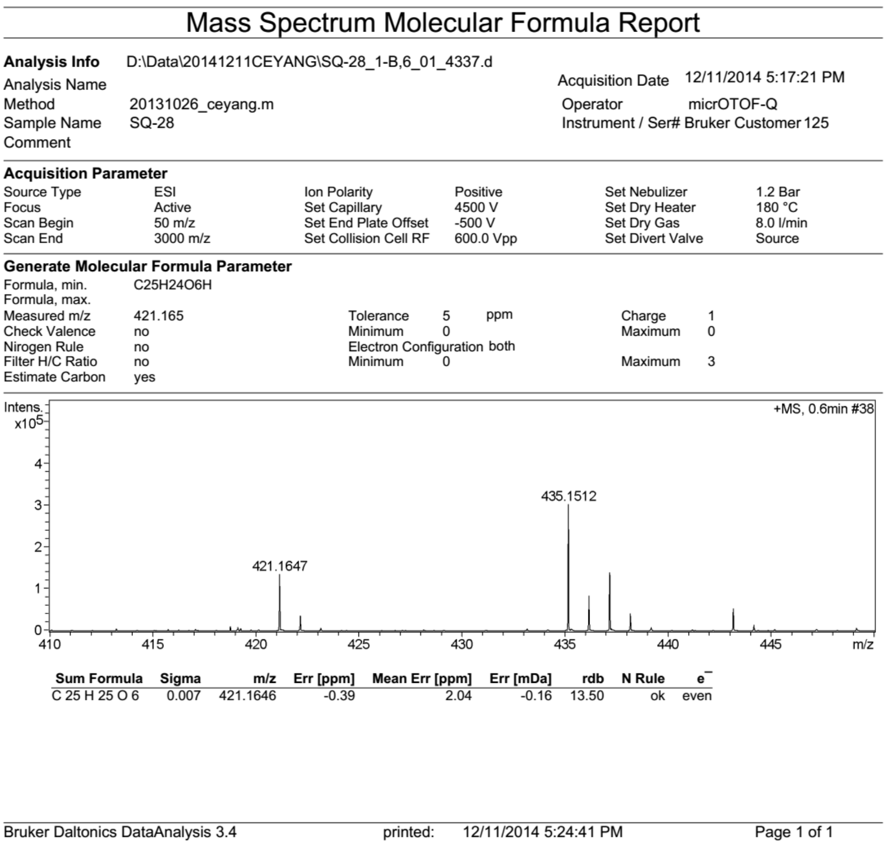

Mass Spectrum Molecular Formula Report

Analysis Info	D:\Data\20141211CEYANG\SQ-28_1-B,6_01_4337.d	Acquisition Date	12/11/2014 5:17:21 PM
Analysis Name			
Method	20131026_ceyang.m	Operator	micrOTOF-Q
Sample Name	SQ-28	Instrument / Ser#	Bruker Customer 125
Comment			

Acquisition Parameter

Source Type	ESI	Ion Polarity	Positive	Set Nebulizer	1.2 Bar
Focus	Active	Set Capillary	4500 V	Set Dry Heater	180 °C
Scan Begin	50 m/z	Set End Plate Offset	-500 V	Set Dry Gas	8.0 l/min
Scan End	3000 m/z	Set Collision Cell RF	600.0 Vpp	Set Divert Valve	Source

Generate Molecular Formula Parameter

Formula, min.	C25H24O6H				
Formula, max.					
Measured m/z	421.165	Tolerance	5 ppm	Charge	1
Check Valence	no	Minimum	0	Maximum	0
Nirogen Rule	no	Electron Configuration	both		
Filter H/C Ratio	no	Minimum	0	Maximum	3
Estimate Carbon	yes				

Sum Formula	Sigma	m/z	Err [ppm]	Mean Err [ppm]	Err [mDa]	rdb	N Rule	e⁻
C 25 H 25 O 6	0.007	421.1646	-0.39	2.04	-0.16	13.50	ok	even

Bruker Daltonics DataAnalysis 3.4 printed: 12/11/2014 5:24:41 PM Page 1 of 1

Supplementary Figure S3.1 HRESIMS spectrum of morusin

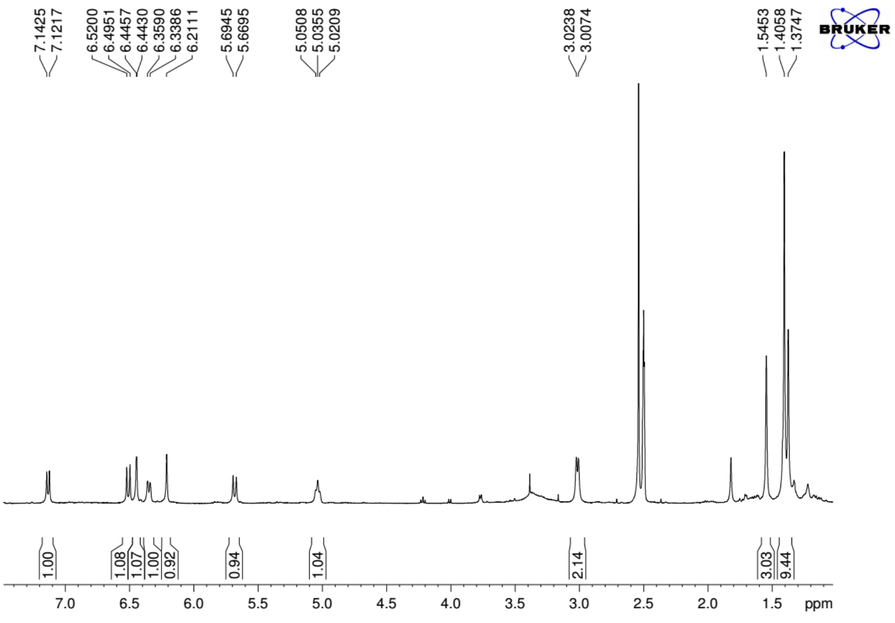

Supplementary Figure S3.2 ^{1}H NMR spectrum (400 MHz, DMSO-d_6) of morusin

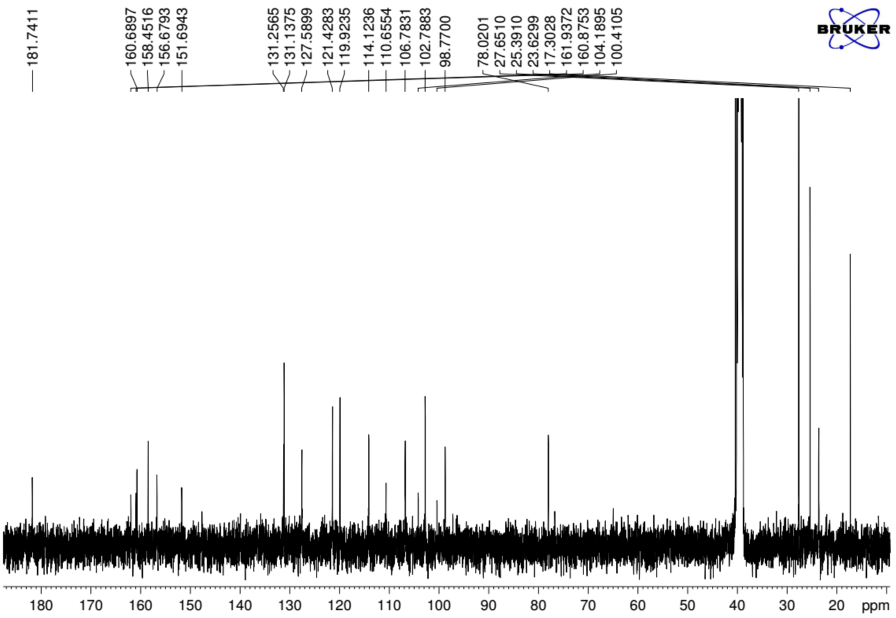

Supplementary Figure S3.3 ^{13}C NMR spectrum (100 MHz, DMSO-d_6) of morusin

Mass Spectrum Molecular Formula Report

Analysis Info

Analysis Name	D:\Data\20150521CEYANG\SQ-27_1-B,2_01_5170.d	Acquisition Date	5/21/2015 11:24:29 AM
Method	20150520yezhi.m	Operator	micrOTOF-Q
Sample Name	SQ-27	Instrument / Ser#	Bruker Customer 125
Comment			

Acquisition Parameter

Source Type	ESI	Ion Polarity	Positive	Set Nebulizer	1.2 Bar
Focus	Active	Set Capillary	4500 V	Set Dry Heater	180 °C
Scan Begin	50 m/z	Set End Plate Offset	-500 V	Set Dry Gas	8.0 l/min
Scan End	3000 m/z	Set Collision Cell RF	200.0 Vpp	Set Divert Valve	Source

Generate Molecular Formula Parameter

Formula, min.	C26H26O7H						
Formula, max.							
Measured m/z	451.175	Tolerance	5	ppm		Charge	1
Check Valence	no	Minimum	0			Maximum	0
Nirogen Rule	yes	Electron Configuration	both				
Filter H/C Ratio	yes	Minimum	0			Maximum	3
Estimate Carbon	yes						

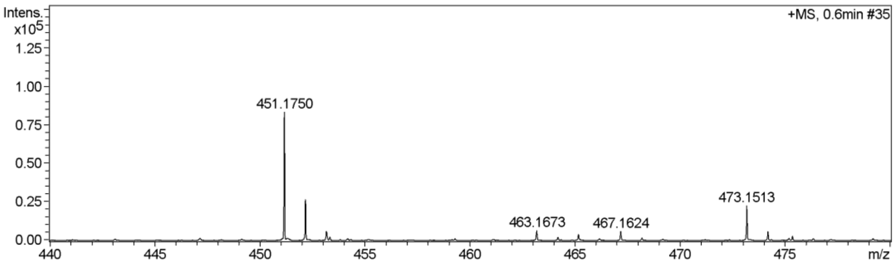

Sum Formula	Sigma	m/z	Err [ppm]	Mean Err [ppm]	Err [mDa]	rdb	N Rule	e⁻
C 26 H 27 O 7	0.021	451.1751	0.23	3.21	0.10	13.50	ok	even

Bruker Daltonics DataAnalysis 3.4 printed: 5/21/2015 11:24:29 AM Page 1 of 1

Supplementary Figure S4.1 HRESIMS spectrum of daphnegiravone A

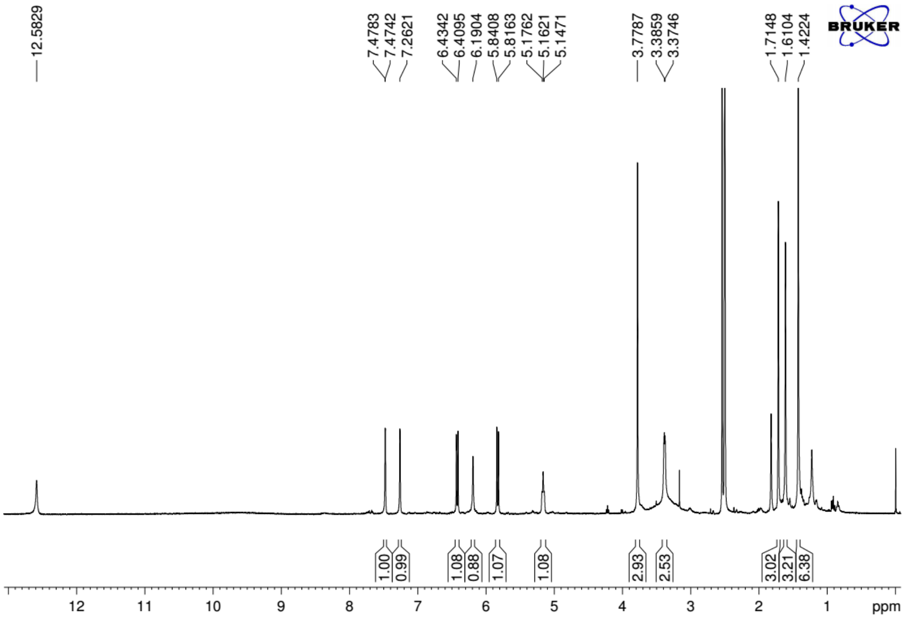

Supplementary Figure S4.2 ^{1}H NMR spectrum (400 MHz, DMSO-d_6) of daphnegiravone A

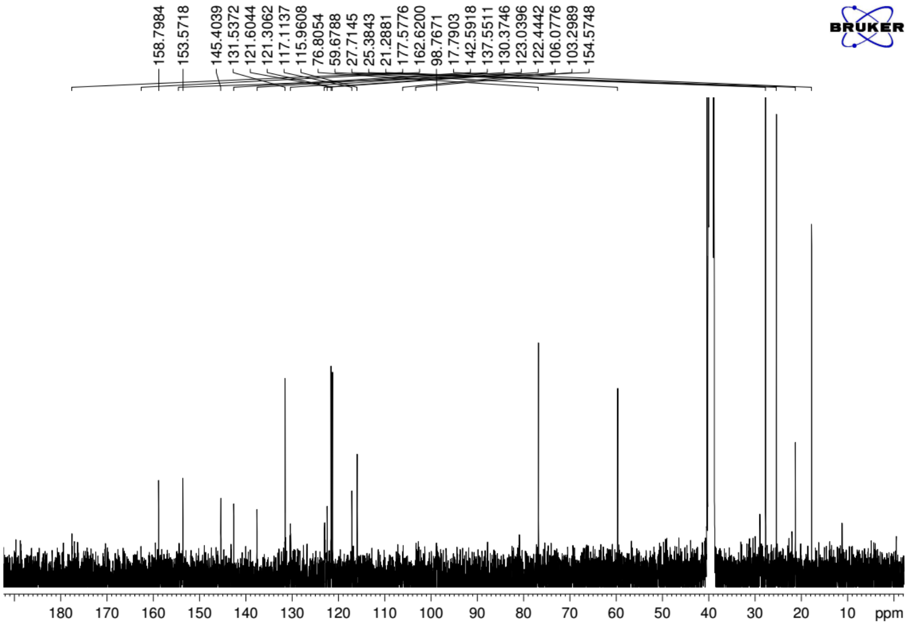

Supplementary Figure S4.3 ^{13}C NMR spectrum (100 MHz, DMSO-d_6) of daphnegiravone A

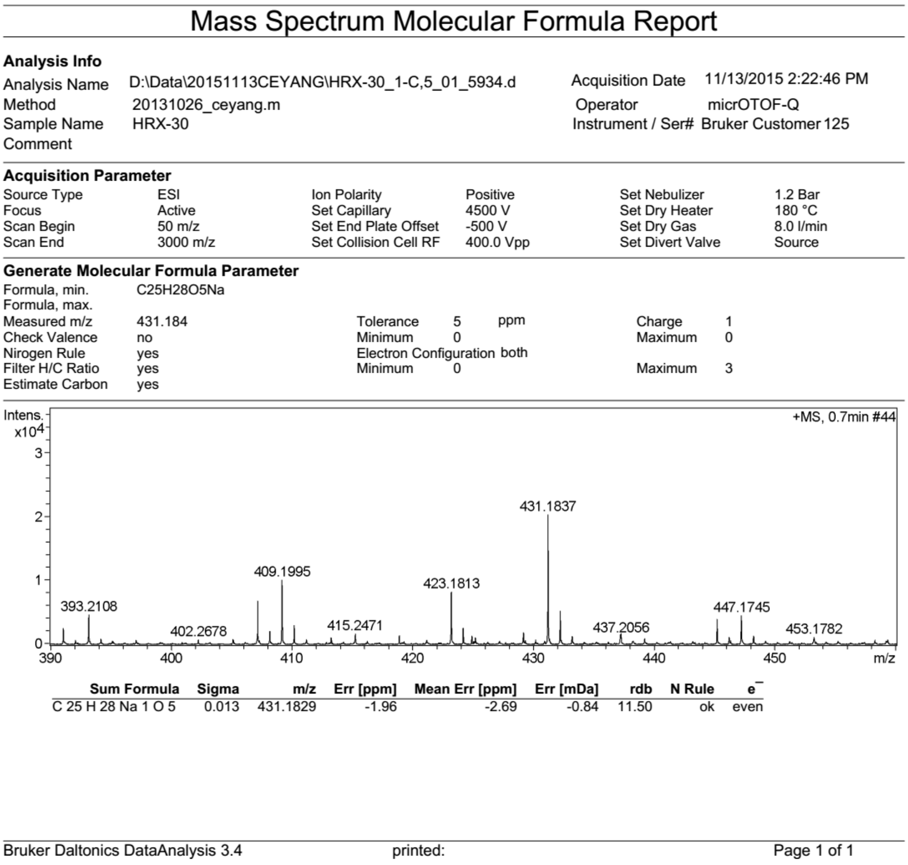

Mass Spectrum Molecular Formula Report

Analysis Info

Analysis Name	D:\Data\20151113CEYANG\HRX-30_1-C,5_01_5934.d	Acquisition Date	11/13/2015 2:22:46 PM
Method	20131026_ceyang.m	Operator	micrOTOF-Q
Sample Name	HRX-30	Instrument / Ser#	Bruker Customer 125
Comment			

Acquisition Parameter

Source Type	ESI	Ion Polarity	Positive	Set Nebulizer	1.2 Bar
Focus	Active	Set Capillary	4500 V	Set Dry Heater	180 °C
Scan Begin	50 m/z	Set End Plate Offset	-500 V	Set Dry Gas	8.0 l/min
Scan End	3000 m/z	Set Collision Cell RF	400.0 Vpp	Set Divert Valve	Source

Generate Molecular Formula Parameter

Formula, min.	C25H28O5Na				
Formula, max.					
Measured m/z	431.184	Tolerance	5 ppm	Charge	1
Check Valence	no	Minimum	0	Maximum	0
Nirogen Rule	yes	Electron Configuration	both		
Filter H/C Ratio	yes	Minimum	0	Maximum	3
Estimate Carbon	yes				

Sum Formula	Sigma	m/z	Err [ppm]	Mean Err [ppm]	Err [mDa]	rdb	N Rule	e⁻
C 25 H 28 Na 1 O 5	0.013	431.1829	-1.96	-2.69	-0.84	11.50	ok	even

Bruker Daltonics DataAnalysis 3.4 printed: Page 1 of 1

Supplementary Figure S5.1 HRESIMS spectrum of daphnegiravone C

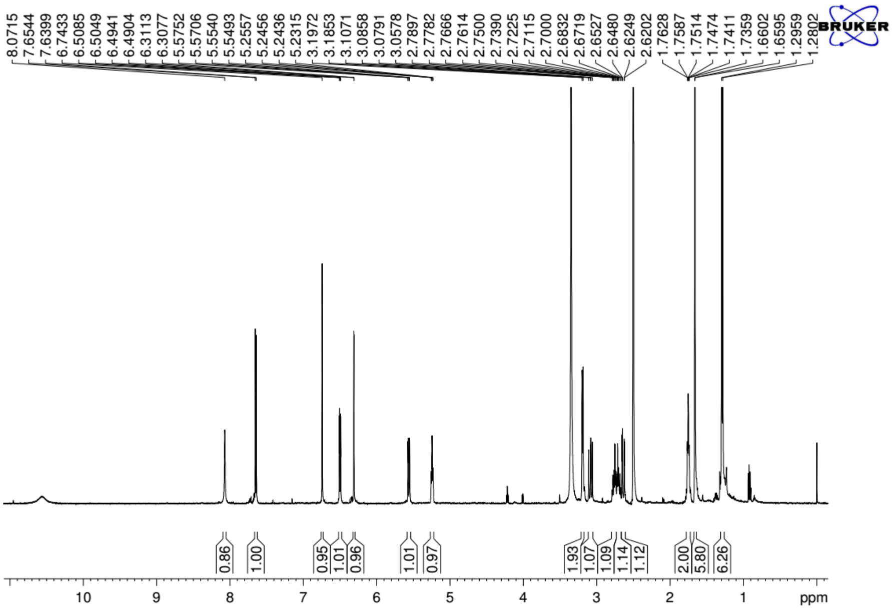

Supplementary Figure S5.2 ^{1}H NMR spectrum (400 MHz, DMSO-d_6) of daphnegiravone C

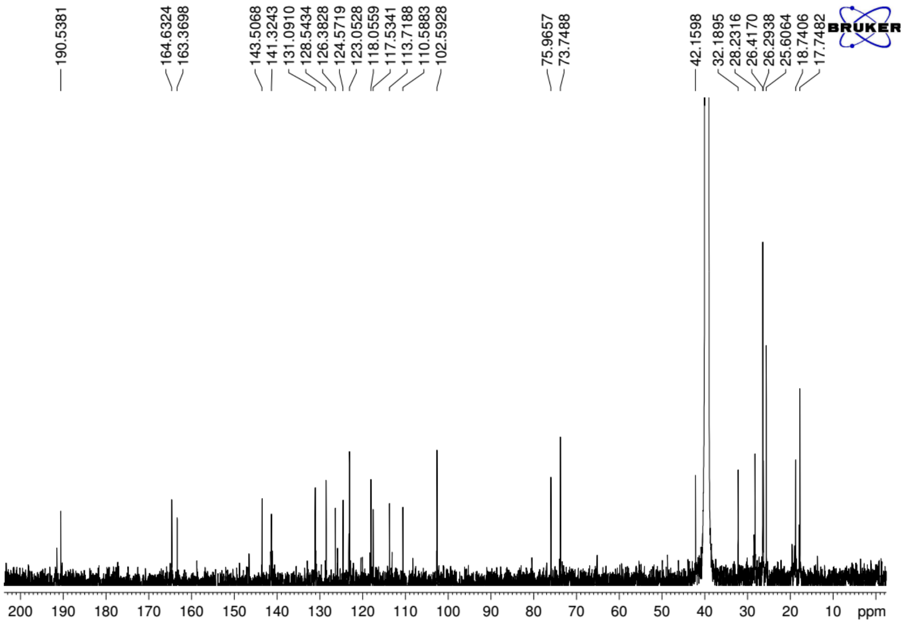

Supplementary Figure S5.3 ^{13}C NMR spectrum (100 MHz, DMSO-d_6) of daphnegiravone C

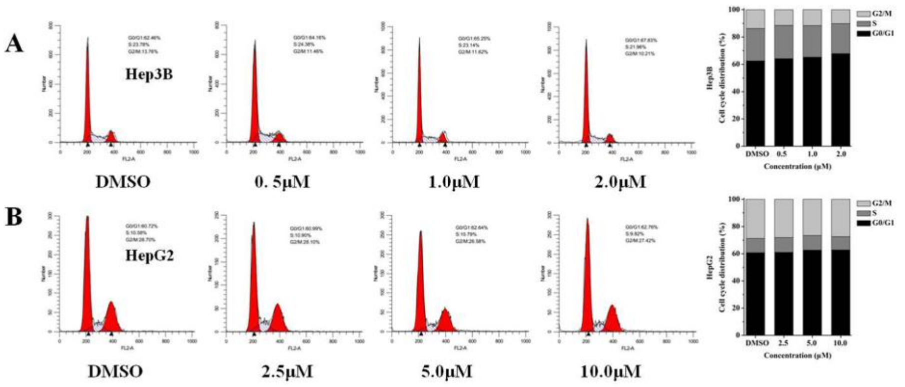

Supplementary Figure S6 Cell cycle effects of daphnegiravone D on Hep3B and HepG2 cells. Cells were induced with a variety of concentrations (0.5–2.0 μM for Hep3B and 2.5–10.0 μM for HepG2 cells) for 24 h, and subsequently cells were harvested, stained and cell cycle were analyzed by flow cytometry. The histograms were presented to describe the effect of cell cycle.

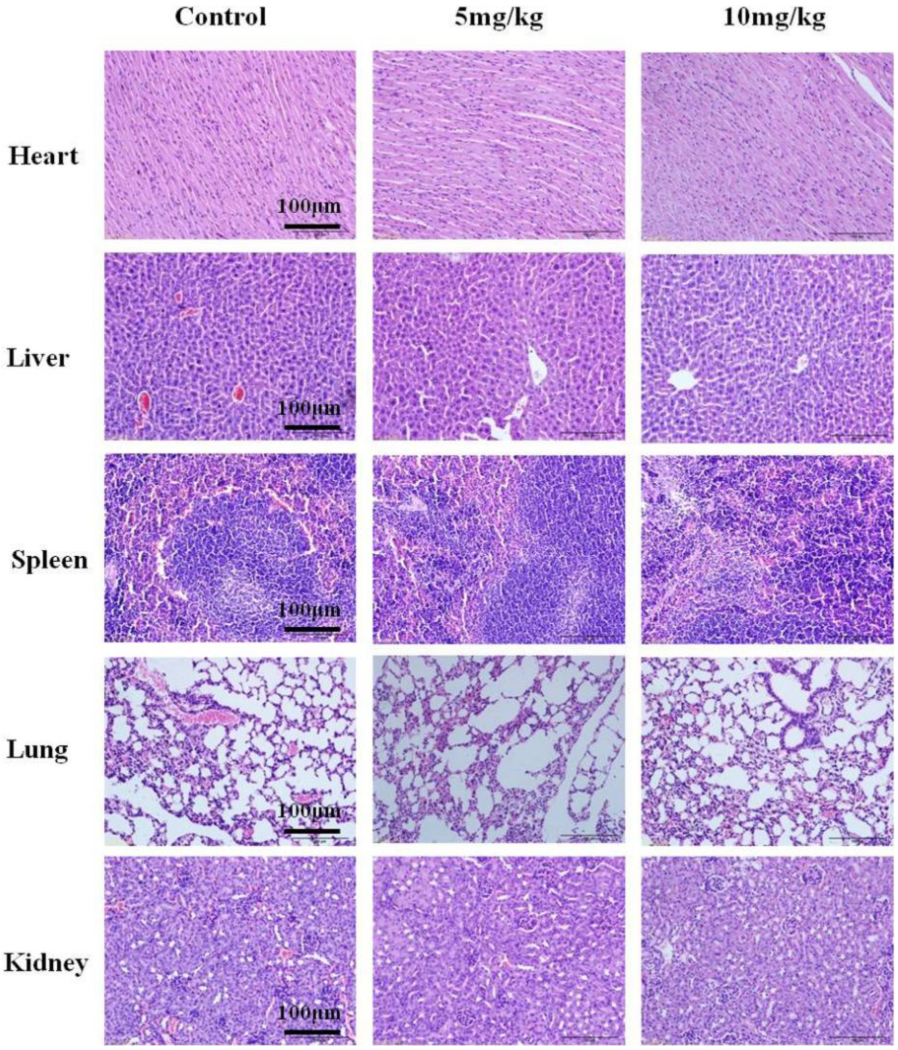

Supplementary Figure S7. Histological analysis of heart, liver, spleen, lung and kidney obtained from nude mice after daphnegiravone D treatment. Hematoxylin-eosin staining; Scale bar, 100 μm.
